# Pathological triad of perioperative acute kidney injury: renal microcirculatory hypoxia, mitochondrial damage, and immuno-metabolic reprogramming

**DOI:** 10.3389/fimmu.2026.1843391

**Published:** 2026-06-08

**Authors:** Shenghua Li, Xiyan Wei, Xudong Han, Jingli Wang, Na Li, Jing Liu, Meiling Zhang

**Affiliations:** Department of Anesthesiology and Surgery, Gansu Provincial Maternal and Child Health Hospital, Gansu Provincial Central Hospital, Lanzhou, Gansu, China

**Keywords:** immunometabolic reprogramming, mitochondrial injury, pathological triangle model, perioperative acute kidney injury, renal microcirculatory hypoxia, sterile inflammation

## Abstract

Perioperative acute kidney injury (AKI) is one of the common and burdensome complications following surgical procedures. The traditional “prerenal” model centered on systemic hemodynamic disturbances fails to adequately account for occult hypoxia that can occur despite relatively stable macro-parameters, as well as significant clinical heterogeneity. Recent clinical and translational studies suggest that perioperative AKI is better characterized as a syndrome involving imbalances in local microcirculation, cellular energy metabolism, and the immune-inflammatory network. Although perioperative AKI is not a typical autoinflammatory disease, the sterile inflammation driven by DAMPs shares common mechanistic features with autoinflammatory syndromes, such as NLRP3 inflammasome activation and its mediated inflammatory amplification. Based on this, this article proposes a “renal microcirculatory hypoxia–mitochondrial injury–immunometabolic reprogramming” pathological triangle model as a conceptual framework for understanding the occurrence, development, and heterogeneity of perioperative AKI. This model emphasizes the shifting dominance of these three components across distinct time windows, as well as their coupled nature and mutually amplifying positive feedback loops. On this basis, this article further discusses imbalance phenotype hypotheses, including microcirculation-dominant, mitochondria-dominant, and immunometabolism-dominant types, and proposes a multidimensional stratification approach corresponding to the pathological triangle model, focusing on renal microcirculation/tissue oxygenation assessment, mitochondrial-related biomarkers, and immunometabolic readouts. Additionally, potential time-windowed intervention pathways are outlined, ranging from preoperative risk optimization, intraoperative perfusion and oxygen delivery management, to postoperative mitochondrial protection and immunometabolic regulation. This article aims to provide a more integrated pathophysiological framework for the mechanistic classification, risk stratification, and multi-target intervention strategies for perioperative AKI.

## 1 Introduction

Perioperative acute kidney injury (AKI) is a common and clinically burdensome complication following surgical procedures and anesthesia, which can significantly increase mortality, prolong hospital stays, and promote the progression of chronic kidney disease (CKD) (1, 2). Despite continuous improvements in perioperative management, AKI remains relatively common after high-risk procedures such as cardiac surgery (3). Its occurrence is influenced by multiple factors, including age, underlying diseases, type of surgery, fluid management, anemia, hyperglycemia, blood product exposure, and nephrotoxic drug exposure, suggesting that perioperative AKI is not driven by a single mechanism (4).

Traditional models primarily focus on “prerenal” hypoperfusion caused by systemic hemodynamic disturbances, but this paradigm has become inadequate in fully explaining the phenomenon of AKI occurring even when systemic hemodynamic parameters are relatively stable (5, 6). Although perioperative hypotension is associated with an increased risk of AKI (7), simply increasing or individually adjusting mean arterial pressure does not always significantly reduce the incidence of perioperative AKI (8). This indicates that relying solely on systemic indicators such as macroscopic blood pressure is still insufficient for comprehensively identifying and preventing perioperative AKI. Clinically, more attention needs to be paid to the local renal microenvironment, particularly microcirculatory and tissue oxygenation abnormalities that are difficult to detect with conventional monitoring.

In recent years, increasing evidence suggests that perioperative AKI is more akin to a network injury process involving local microcirculation, cellular energy metabolism, and immune-inflammatory responses (9). Among these, renal microcirculatory hypoxia is considered one of the important upstream triggers (10, 11). Observational studies suggest that higher oxygen exposure may be associated with an increased risk of postoperative organ injury, but the direct link between this and abnormal renal microcirculatory oxygenation remains to be further elucidated (12). Simultaneously, mitochondrial damage and innate immune activation induced by perioperative stress may also collectively participate in the occurrence and amplification of AKI (13). It should be noted that perioperative AKI is not a classic autoinflammatory disease; however, the sterile inflammation triggered by surgical trauma, ischemia-reperfusion, mitochondrial damage, and DAMPs release shares several mechanistic features with autoinflammatory syndromes, particularly the activation of the NLRP3 inflammasome and IL-1β/IL-18-related inflammatory amplification (14). This mechanistic overlap also supports discussing the pathological process of perioperative AKI from the perspectives of immune inflammation and autoinflammation.

**Figure 1:**
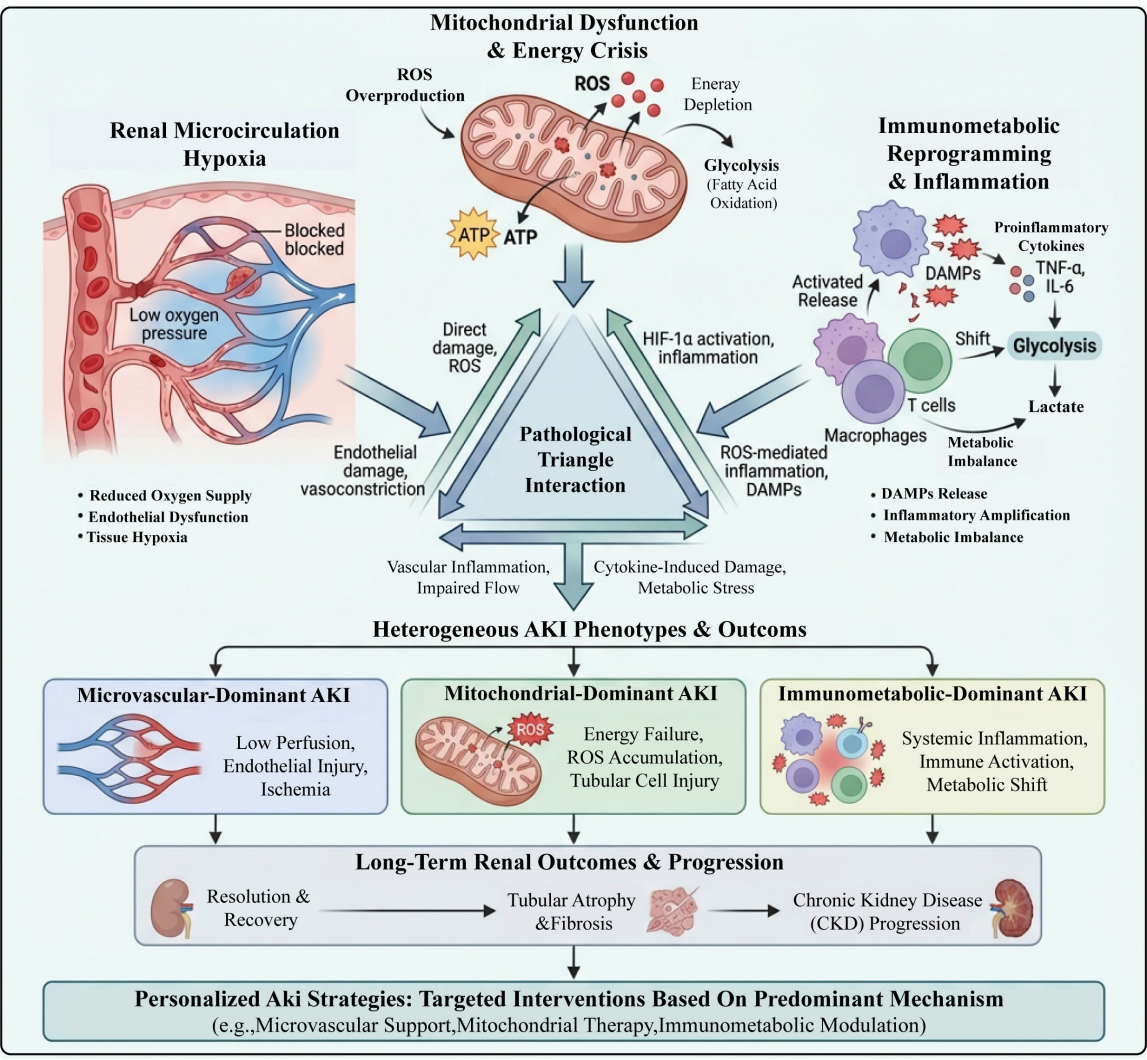
Pathological triangle model and heterogeneity mechanisms of perioperative AKI. This image categorizes perioperative acute kidney injury into three interconnected core pathological dimensions: renal microcirculatory hypoxia, mitochondrial dysfunction and energy crisis, and immune-metabolic reprogramming and inflammation. These three dimensions mutually amplify through ROS, DAMPs, cytokines, and metabolic stress, forming a positive feedback network within the pathological triangle model, ultimately driving different AKI phenotypes, including microcirculation-dominant, mitochondria-dominant, and immune-metabolic-dominant types. The aforementioned heterogeneous phenotypes dictate patient outcomes, including recovery, persistent tubular atrophy and fibrosis, or progression to chronic kidney disease, thus suggesting the need for individualized intervention based on the dominant mechanism, such as microcirculatory support, mitochondrial protection, and immune-metabolic regulation.

Building on these insights, we propose a pathological triangle model consisting of “renal microcirculatory hypoxia–mitochondrial damage–immunometabolic reprogramming.” In this context, ‘immunometabolic reprogramming’ denotes the coordinated metabolic shifts in immune and renal parenchymal cells during perioperative AKI, including enhanced glycolysis, suppressed oxidative phosphorylation/fatty acid oxidation (OXPHOS/FAO), abnormal mitochondrial signaling, and cytokine-mediated inflammatory amplification, which may be involved in the maintenance of sterile inflammation and failure of repair. Notably, microcirculatory dysfunction, mitochondrial damage, and inflammation/immunometabolic abnormalities are not entirely new mechanisms. Previous AKI research, particularly studies on sepsis-associated AKI and ischemia-reperfusion injury, has extensively discussed these aspects from the perspectives of hemodynamic abnormalities, renal tubular stress, mitochondrial dysfunction, and inflammation–metabolism crosstalk. The pathological triangle model proposed in this article does not aim to replace these existing frameworks but rather attempts to integrate them into the specific temporal window, combined insults, and clinical heterogeneity context of perioperative AKI. This model posits that these three components are interdependent, forming a self-reinforcing positive feedback loop via mutual coupling. By exhibiting distinct dominant imbalances across patients, this framework offers a time-resolved and stratification-oriented approach to explaining the clinical heterogeneity of perioperative AKI. This article will accordingly review the key mechanisms, phenotypic heterogeneity, and their potential implications for stratified management of perioperative AKI from the three levels of organ, organelle, and immune–metabolic network (**Figure 1**).

## 2 Perioperative renal microcirculatory hypoxia: an upstream trigger in the pathological triangle model

### 2.1 Renal microcirculatory anatomy and oxygen consumption characteristics: supply-demand imbalance in the cortex and medulla

Although the kidney receives abundant blood flow, there is a distinct regional difference in oxygen distribution: the cortex has relatively sufficient blood supply, while the medulla, especially the inner stripe of the outer medulla, is in a state of physiological hypoxia for an extended period (15, 16). This feature is primarily attributed to the high energy demand of active sodium reabsorption in the thick ascending limb of the loop of Henle, along with the oxygen diffusion limitation caused by countercurrent exchange in the vasa recta, making the medulla particularly sensitive to fluctuations in blood flow and oxygen supply (17). Perioperative hypoperfusion, inflammation, and reduced oxygen delivery can rapidly disrupt this fragile balance, shifting the medulla from physiological hypoxia to pathological hypoxia, potentially serving as an early vulnerable link in perioperative AKI (18, 19). Although direct evidence from perioperative populations remains limited, models of endotoxic shock, diabetes, and other renal injuries have shown that the deep cortex and outer medulla are more prone to decreased oxygenation (20, 21). These findings provide indirect mechanistic support for the hypothesis that perioperative renal microcirculatory hypoxia occurs, but their applicability in real-world perioperative populations requires further validation through direct microcirculatory and tissue oxygenation monitoring studies.

### 2.2 Effects of common perioperative insults on renal microcirculation

Perioperative renal microcirculatory imbalance is typically driven by multiple factors, including hypotension, hypovolemia, hemodilution, cardiopulmonary bypass, anesthetics, and vasoactive agents (22, 23). Anesthesia and sedative drugs may affect renal blood flow autoregulation by altering autonomic tone and vascular reactivity, thereby exacerbating susceptibility to medullary hypoxia (24). For example, a randomized controlled trial in kidney transplant patients showed that perioperative dexmedetomidine reduced early postoperative creatinine levels but did not significantly improve total vascular density in the sublingual microcirculation, suggesting that the net effect of anesthesia-related interventions on microcirculation may vary across tissues, and its impact on renal medullary oxygenation requires further investigation (25, 26).

Hypovolemia, blood pressure fluctuations, and vasoactive agents are more direct hemodynamic factors. When effective circulating blood volume is insufficient or mean arterial pressure falls below the lower limit of renal autoregulation, renal perfusion may significantly decline, whereas agents like norepinephrine may, under certain conditions, exacerbate renal vasoconstriction and regional perfusion heterogeneity (27, 28). It should be noted that although a stable observational association exists between perioperative hypotension and increased AKI risk, raising or individualizing mean arterial pressure (MAP) targets has not consistently reduced AKI incidence across clinical trials (29). This inconsistency suggests that macro-level blood pressure does not fully represent renal microcirculatory perfusion and medullary oxygenation status, also indicating the limited translational effect of purely hemodynamic optimization.

Severe hemodilution can result in a dissociation between “adequate macro-perfusion and insufficient tissue oxygen supply”; porcine models show that when hematocrit drops to 10%, injury markers such as NGAL are elevated even if apparent perfusion is maintained (30). Cardiopulmonary bypass and ischemia-reperfusion during cardiac surgery can further trigger inflammation, hemolysis, endothelial injury, and microthrombus formation, leading to local “no-reflow” and exacerbating renal tissue hypoxia (31, 32). Additionally, observational studies suggest that higher oxygen exposure may be associated with increased risk of postoperative organ injury, but the causal relationship and specific effects on renal microcirculatory oxygenation remain to be validated (33). Therefore, perioperative renal microcirculatory injury should not be solely attributed to abnormalities in macro-level blood pressure or cardiac output, nor should it be assumed that raising MAP, increasing oxygen exposure, or intensifying fluid therapy will necessarily improve renal medullary oxygenation; it is more likely the result of combined effects of volume status, oxygen-carrying capacity, vascular tone, drug effects, and reperfusion stress.

### 2.3 Microcirculatory dysfunction and tissue hypoxia: from normal macro-perfusion to “hidden hypoxia”

A key feature of perioperative AKI is “hidden hypoxia,” where even after systemic blood pressure, cardiac output, and other macro-hemodynamic parameters have been corrected, the renal microcirculation may remain functionally or structurally abnormal (34). In hemorrhagic shock models, although fluid resuscitation restores systemic blood pressure and apparent renal blood flow, contrast-enhanced ultrasound (CEUS) still demonstrates abnormal renal cortical perfusion patterns, accompanied by persistent hyperlactatemia and elevated NGAL (35). In sepsis animal models, renal cortical perfused vessel density and microvascular flow index decrease, while hypoxia probe-positive cells increase (36, 37). Although these studies primarily come from non-perioperative models, they consistently suggest that restoration of macro-perfusion does not equate to recovery of renal tissue oxygenation, offering an indirect mechanistic basis for understanding how AKI can progress even after perioperative hypotension is corrected.

This “macro-normal, micro-imbalanced” phenomenon may be driven by endothelial dysfunction, increased heterogeneity in capillary perfusion, decreased functional capillary density, and leukocyte adhesion, leading to increased oxygen diffusion distance and reduced tissue oxygen utilization efficiency (38). Therefore, if early identification of perioperative AKI still relies only on macro-circulatory indicators, it may fail to timely capture the actual hypoxic process occurring locally in the kidney. This also helps explain why some perioperative hemodynamic optimization strategies, higher MAP targets, or oxygen delivery regulation approaches have not consistently shown renal protective effects in clinical studies: although these interventions can improve systemic circulatory readings, they may not simultaneously restore renal medullary microcirculation, oxygen diffusion efficiency, and cellular oxygen utilization status.

In this context, technologies such as NIRS, BOLD-MRI, and bedside CEUS are being explored for non-invasive assessment of renal tissue oxygenation (39). However, these methods are currently mostly research or early translational tools, with evidence primarily from non-perioperative populations, positioning them as promising avenues for microcirculatory assessment in perioperative AKI rather than established routine clinical tools. It should be emphasized that current evidence for “restoration of macro-perfusion not equating to recovery of renal tissue oxygenation” mainly comes from shock, sepsis, or other non-perioperative models; its applicability in perioperative AKI remains a reasonable extrapolation and requires validation through direct microcirculatory and tissue oxygenation monitoring studies.

### 2.4 How microcirculatory hypoxia “lays the groundwork” for mitochondrial injury and immune-metabolic imbalance

**Figure 2:**
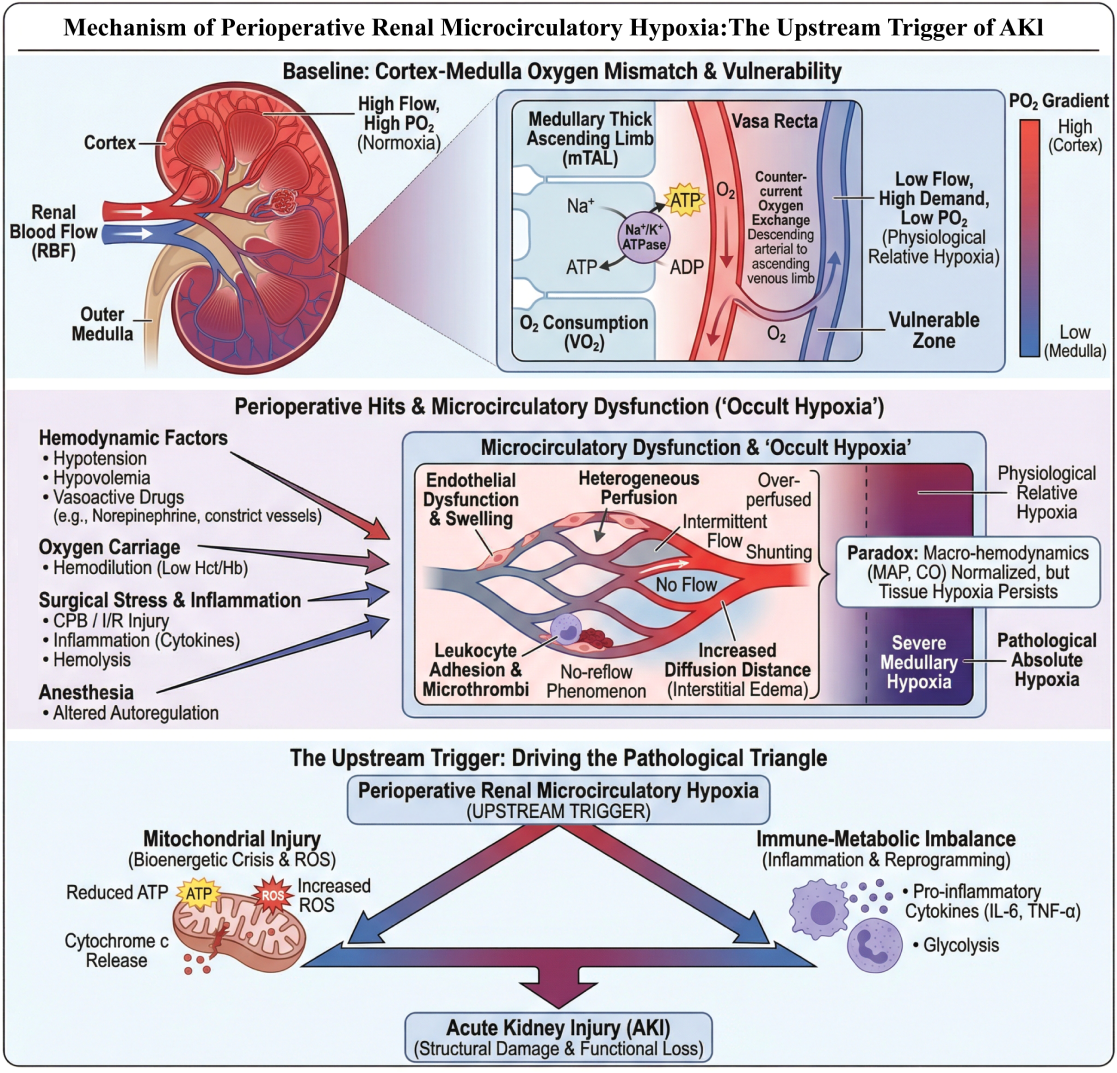
Perioperative renal microcirculatory hypoxia: the upstream trigger in the pathological triangle model. The renal cortex–medulla inherently exhibits a mismatch between oxygen supply and consumption, with the outer medulla characterized by physiological relative hypoxia due to low blood flow and high oxygen consumption. Multiple perioperative insults, such as hypotension, hypovolemia, hemodilution, inflammation, ischemia-reperfusion, and anesthesia, may precipitate endothelial injury, perfusion heterogeneity, shunting, microthrombi, and interstitial edema, leading to “covert hypoxia.” Even when systemic hemodynamic parameters normalize, local tissue hypoxia may persist. In the pathological triangle model, renal microcirculatory hypoxia, as the earliest upstream trigger, can propagate mitochondrial damage, ROS accumulation, and immunometabolic imbalance, ultimately culminating in structural damage and functional loss in perioperative AKI.

Microcirculatory hypoxia in perioperative AKI acts more as an upstream triggering factor than an isolated endpoint. On one hand, it reshapes the spatial oxygen gradient in the kidney, pushing the outer medulla, already in a “high energy demand-low oxygen reserve” state, closer to the energy supply limit; on the other hand, increased perfusion heterogeneity and the formation of focal no-reflow zones expose local tissues to hypoxia and metabolic stress earlier (40). Therefore, renal microcirculatory hypoxia is better understood as a pivotal initiating event for subsequent mitochondrial crisis and inflammatory amplification, rather than a standalone pathological phenomenon. The focus of this chapter is not to elaborate on all downstream pathways in advance, but to emphasize that microcirculatory hypoxia is the earliest identifiable and most promising upstream trigger for early intervention in the pathological triangle model. How it subsequently drives mitochondrial dysfunction and immune-metabolic reprogramming will be discussed in Chapters 3 and 4 (**Figure 2**).

## 3 Mitochondrial damage: the energy metabolism dimension connecting hypoxia and cell fate

### 3.1 The particularity of renal mitochondria: proximal tubules, high energy consumption, and susceptibility

The kidney is an organ highly dependent on energy metabolism. Its energy demands primarily come from the renal tubules, especially the active reabsorption function of the proximal tubules (PT) (41). Proximal tubule epithelial cells (PTECs) are rich in mitochondria and need to process over 99% of the glomerular filtrate, making them highly dependent on OXPHOS and FAO (42). This metabolic characteristic makes PTECs particularly sensitive to hypoxia, acidosis, reperfusion stress, and mitochondrial quality control disorders, making them more prone to energy imbalance and structural damage in conditions such as ischemia-reperfusion injury (IRI) in AKI (43, 44). Although PTECs have a certain degree of metabolic flexibility and can switch between FAO, glycolysis, and glutamine metabolism to adapt to stress, in AKI, especially sepsis-associated AKI, this flexibility is often disrupted, manifested as decreased mitochondrial oxygen consumption, inhibited biogenesis, reduced fusion, decreased expression of FAO enzymes, and reduced ATP synthesis efficiency (45–47). Furthermore, modeling studies suggest that compared to the thick ascending limb cells, which are more adapted to hypoxia, PTECs have more difficulty maintaining ATP production when mitochondrial oxygen partial pressure is extremely low (48). Therefore, PTECs can be considered the most vulnerable energy hub in perioperative AKI; once renal microcirculatory hypoxia affects this cell population, local oxygen supply abnormalities can be amplified into an energy crisis at the mitochondrial level.

### 3.2 Mitochondrial structural and functional dysfunction under ischemia-reperfusion

Ischemia-reperfusion can damage the mitochondrial electron transport chain (ETC), inhibit the function of complexes I, IV, and V, leading to insufficient ATP synthesis, failure of Na^+^/K^+^-ATPase, increased intracellular Na^+^/Ca²^+^ load, and the promotion of cell swelling, necrosis, or programmed death (49–51). For perioperative AKI, this means that even if macroscopic perfusion is corrected, residual ETC inhibition and ion homeostasis disorders during the reperfusion phase may still promote the progression of renal tubular damage. ETC dysfunction can also induce an increase in mitochondrial ROS (mtROS) through electron leakage. Damage to complexes I/III and succinate accumulation during the ischemic phase can promote the generation of superoxide anions and hydrogen peroxide during the reperfusion phase (52, 53). Excessive ROS can cause cardiolipin oxidation, disruption of membrane integrity, and mtDNA damage, further aggravating ETC dysfunction (54, 55). Although mitochondrial-targeted antioxidants have shown protective effects in various animal IRI models, their clinical translation remains unstable, suggesting that mtROS is an important candidate target but cannot be simply equated with a validated therapeutic entry point (56). As calcium overload, increased ROS, and ATP depletion accumulate, mitochondrial damage can further converge on the opening of the mitochondrial permeability transition pore (mPTP) (57). Sustained opening of mPTP leads to membrane potential collapse, matrix swelling, outer membrane rupture, and cytochrome c release, thereby inducing apoptosis or necrosis (58, 59). Current evidence for mPTP-related interventions partly comes from myocardial or other non-renal models, so in perioperative AKI, it should still be considered a reasonable extrapolation and candidate mechanism. Overall, multiple exposures during the perioperative period may collectively precipitate a renal tubular energy crisis through reperfusion stress, microcirculatory heterogeneity, and mitochondrial susceptibility, but direct clinical evidence linking these mechanisms remains limited.

### 3.3 Mitochondrial quality control: imbalance of fusion/fission, mitophagy, and biogenesis

Under physiological conditions, mitochondria maintain network stability through fusion/fission, mitophagy, and biogenesis (60). Mfn1/2 and OPA1 mediate fusion, Drp1 mediates fission, the PINK1/Parkin pathway clears damaged mitochondria, and the PGC-1α/NRF1/TFAM axis promotes new mitochondrial generation (61–64). Under AKI stress, this quality control system frequently displays a triad of imbalances characterized by excessive fission, insufficient clearance, and inhibited biogenesis. Drp1-mediated enhanced fission, reduced levels of fusion proteins such as Mfn2, impaired mitophagy, and PGC-1α inhibition can collectively result in mitochondrial fragmentation, accumulation of abnormal mitochondria, increased ROS, and decreased energy production (65, 66). This pattern has been repeatedly observed in models such as IRI, diabetic nephropathy, and drug-induced kidney injury, suggesting that mitochondrial quality control disorders are an important link for renal cells to transition from reversible dysfunction to death or repair failure.

### 3.4 Release of mitochondrial DAMPs: mtDNA, cardiolipin, etc., as immune activation signals

When mitochondria are severely damaged or membrane structure is disrupted, they can release mitochondrial-derived damage-associated molecular patterns (mtDAMPs), such as mtDNA, cardiolipin, and ATP. Due to retaining some “bacteria-like” molecular characteristics, mtDAMPs may activate the innate immune system (67, 68). In the pathological triangle model, mtDAMPs can be considered candidate bridge signals connecting mitochondrial damage and immune activation, transforming an energy crisis confined within cells into a sterile inflammatory response (69, 70). mtDNA is one of the most studied mtDAMPs, which can induce type I interferon and inflammatory factor expression through the cGAS–STING or TLR9 pathways (71). This axis has been supported by more evidence in sepsis, autoimmune diseases, and chronic inflammatory states (72, 73). In addition to mtDNA, cardiolipin and ATP are also representative mtDAMPs (74). Cardiolipin may externalize upon inner membrane damage or oxidation, promoting NLRP3 inflammasome assembly and activation; extracellular ATP can further enhance inflammasome-related responses through the P2X7 receptor (75–77). Therefore, mtDNA, cardiolipin, and ATP may together constitute a “mitochondrial–innate immune” alarm system. In the perioperative setting, factors such as surgical trauma, extracorporeal circulation, aortic clamping, blood transfusion, and repeated ischemia-reperfusion can induce mitochondrial damage and consequent mtDAMP release (78). However, it should be noted that direct clinical evidence for the mtDAMPs-cGAS/STING-NLRP3 axis in perioperative AKI is still limited, and current evidence is more based on IRI, sepsis AKI, systemic inflammation, and preclinical studies. Therefore, this article treats it as a candidate mechanism for the conversion of mitochondrial damage to sterile inflammation, rather than a fully validated clinical causal pathway.

### 3.5 The “memory effect” of mitochondrial damage in the progression from acute injury to chronic kidney disease

**Figure 3:**
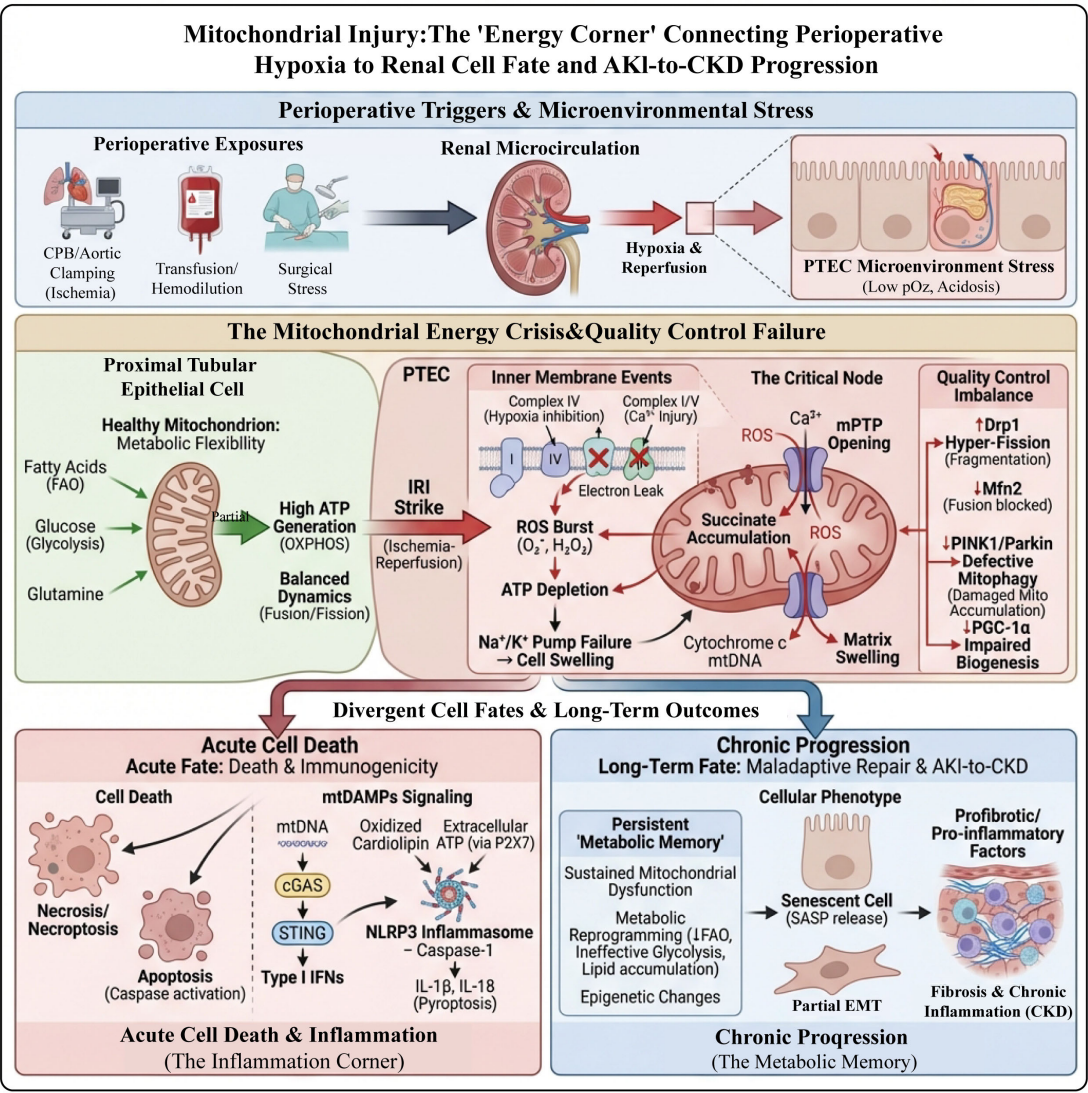
Mitochondrial damage in the pathological triangle model: the “energy hub” linking perioperative hypoxia, AKI onset, and AKI-to-CKD progression. Perioperative ischemia, hemodilution, and surgical stress exert direct effects on proximal tubular epithelial cells through renal microcirculatory hypoxia/reperfusion, triggering inhibition of the mitochondrial electron transport chain, a surge in ROS, ATP depletion, Ca²^+^ overload, and mPTP opening, accompanied by excessive fission, impaired mitophagy, and decreased biogenesis, resulting in an “energy crisis coupled with quality control failure”. Subsequently, cells may follow one of two distinct pathways: first, acute-phase apoptosis, necrosis, or necroptosis, accompanied by the activation of cGAS–STING and NLRP3 inflammasomes via mtDAMPs such as mtDNA, ATP, and oxidized cardiolipin, thereby amplifying the inflammatory response; second, entering a state of persistent mitochondrial dysfunction and “metabolic memory”, inducing senescence, partial EMT, and a profibrotic/proinflammatory phenotype, ultimately driving maladaptive repair, renal fibrosis, and progression from AKI to CKD.

Sublethal mitochondrial damage is considered one of the important mechanisms for the transition from AKI to CKD. Even after the acute insult has been resolved, PTECs may still retain persistent metabolic abnormalities, impaired energy utilization, and stress susceptibility. This phenomenon can be conceptualized through the framework of “metabolic memory” (79). Epidemiological studies show that a single episode of perioperative AKI can increase the risk of long-term CKD and adverse cardiorenal events, suggesting that even after clinical function “recovers,” there may still be a long-term vulnerable state at the cellular and mitochondrial levels in the kidney (80). After AKI, PTECs can exhibit persistent but not necessarily fatal mitochondrial dysfunction, including excessive fission, increased ROS, abnormal membrane potential, and mitochondrial network fragmentation (44). These changes can be perpetuated through metabolic reprogramming and epigenetic regulation, transitioning cells from a transient stress state to a long-term state of inefficient energy supply and reduced repair capacity (81, 82). These changes can be perpetuated through metabolic reprogramming and epigenetic regulation, transitioning cells from a transient stress state to a long-term state of inefficient energy supply and reduced repair capacity (83, 84). Therefore, the core significance of “mitochondrial metabolic memory” lies in the fact that sublethal mitochondrial damage may leave persistent energy utilization defects within PTECs, giving renal tubules a lower recovery threshold and higher injury susceptibility in subsequent stresses. This mechanism provides a mechanistic explanation at the cellular level for how a single episode of perioperative AKI increases the long-term risk of CKD and also suggests that early identification and intervention of mitochondrial damage may help block the AKI–CKD transformation chain (**Figure 3**).

## 4 Immunometabolic reprogramming: the metabolic basis of inflammatory amplification and repair failure

The term immunometabolic reprogramming in this chapter refers to alterations in metabolic programs in immune cells, renal tubular epithelial cells, and endothelial cells following perioperative stress. These changes are primarily manifested as enhanced glycolysis in pro-inflammatory immune cells, suppression of reparative OXPHOS/FAO programs, mtDAMP-mediated innate immune activation, and inflammatory amplification sustained by metabolic intermediates and cytokines. Notably, direct clinical evidence from human populations for this process in perioperative AKI remains limited, with current evidence mainly derived from sepsis AKI, ischemia-reperfusion injury, renal tubular injury models, and inflammatory disease studies. Therefore, this chapter presents it as a candidate amplification mechanism that may exist in perioperative AKI, rather than a fully validated clinical causal chain.

### 4.1 Major immune cell lineages in perioperative AKI

The development of AKI may involve sequential participation of innate and adaptive immunity. Resident kidney macrophages and dendritic cells form the basis of immune surveillance (85); animal studies show that surgical trauma can rapidly activate renal immune cells and induce pro-inflammatory gene expression. In human rhabdomyolysis-associated AKI, infiltration by macrophages, dendritic cells, and T cells is observed around injured renal tubules, accompanied by inflammasome activation and elevated TNF-α, though this evidence cannot be fully equated with perioperative AKI (86). Following perioperative stress and reperfusion, neutrophils and monocytes may be recruited to the kidney. IRI mouse models suggest that cDC1 deficiency can increase neutrophil infiltration and exacerbate renal tubular injury (87). The monocyte-macrophage lineage exhibits plasticity, with M1-like macrophages driving pro-inflammatory responses and M2-like macrophages facilitating repair (88). Additionally, T cell subsets, particularly CD4^+^ and CD8^+^ T cells, participate in regulating the balance between inflammation and repair in the mid-to-late stages of AKI (89). Although not all evidence comes from perioperative populations, it collectively supports a reasonable hypothesis: perioperative AKI may involve a sequential immune remodeling process from rapid innate immune activation to adaptive immune participation, providing a cellular basis for immunometabolic reprogramming.

### 4.2 Basic patterns of immune cell metabolic reprogramming

Immune cell metabolic reprogramming is a core adaptive process when immune cells transition from a resting to an activated state, with metabolic programs highly coupled to functional phenotypes (90). However, it should be noted that systematic immunometabolomics and single-cell multi-omics validation are lacking for perioperative AKI; thus, the following patterns are primarily derived from mechanistic references in acute and chronic inflammation, tumor microenvironments, sepsis AKI, and ischemia-reperfusion models. In general, pro-inflammatory immune cells often exhibit enhanced glycolysis, while repair- or tolerance-associated cells rely more on OXPHOS/FAO. M1 macrophages, Th1/Th17 cells, and neutrophils typically show enhanced glycolysis, accompanied by accumulation of metabolic intermediates such as succinate, which can promote IL-1β and other inflammatory factor expression via HIF-1α, NF-κB, and other mechanisms (91, 92). Conversely, M2 macrophages, regulatory T cells (Tregs), and memory T cells rely more on OXPHOS and FAO (93, 94). Therefore, the “glycolysis-biased pro-inflammatory program” and “OXPHOS/FAO-dominated repair program” can serve as a candidate framework for understanding immunometabolic imbalance in perioperative AKI, rather than validated clinical classification criteria.

### 4.3 How mitochondrial injury drives immunometabolic reprogramming

Severe renal tubular mitochondrial injury can release mtDAMPs such as mtDNA, cardiolipin, and ATP. This section focuses not on reiterating the release mechanisms of mtDAMPs, but on explaining how these signals may contribute to sustained pro-inflammatory immune responses. Initially, innate immune signals triggered by mtDAMPs may induce immune cells to switch from a resting mode dominated by OXPHOS/FAO to a pro-inflammatory program dominated by glycolysis. Enhanced glycolysis not only meets the energy and biosynthetic demands of activation but may also lead to accumulation of metabolic intermediates such as lactate and succinate; these molecules can further promote expression of pro-inflammatory factors like IL-1β and TNF-α by stabilizing HIF-1α and activating NF-κB (95–97). On the other hand, metabolic intermediates and epigenetic reprogramming may contribute to the persistence of this pro-inflammatory program. Abnormal accumulation of metabolites such as lactate and succinate may help maintain expression of glycolysis-related genes and influence the chromatin accessibility of inflammation-related genes through mechanisms like histone lysine lactylation (98). Thus, mitochondrial injury may not only induce transient inflammatory responses but also, through mtDAMPs, metabolic intermediates, and epigenetic reprogramming, predispose immune cells to maintain a pro-inflammatory metabolic state. This mechanistic cascade remains hypothetical based on indirect evidence in perioperative AKI, requiring further validation through single-cell omics, metabolomics, and longitudinal inflammatory marker monitoring in perioperative cohorts.

### 4.4 Paracrine effects of immunometabolic reprogramming on renal tubular epithelial cells and endothelial cells

Evidence for the paracrine effects of immune cell metabolic reprogramming on renal tubules and endothelial cells primarily comes from general AKI and CKD studies. Activated immune cells release TNF-α, IL-1β, IL-6, chemokines, and ROS, which can damage renal tubular epithelial cells and microvascular endothelial cells, disrupt the epithelial barrier and endothelial homeostasis, and further exacerbate microcirculatory dysfunction (99, 100). Simultaneously, this pro-inflammatory microenvironment can drive renal tubular epithelial cells to shift from an efficient OXPHOS-dominated metabolic mode toward glycolysis. In the short term, this facilitates stress adaptation; however, if sustained, ATP production efficiency decreases, impairing active transport and tissue repair, further inhibiting mitochondrial function and rendering cells more susceptible to repair failure or pro-fibrotic states (101, 102).

### 4.5 From acute inflammation to immune scar-like state: bridging immunometabolic imbalance and fibrosis

**Figure 4:**
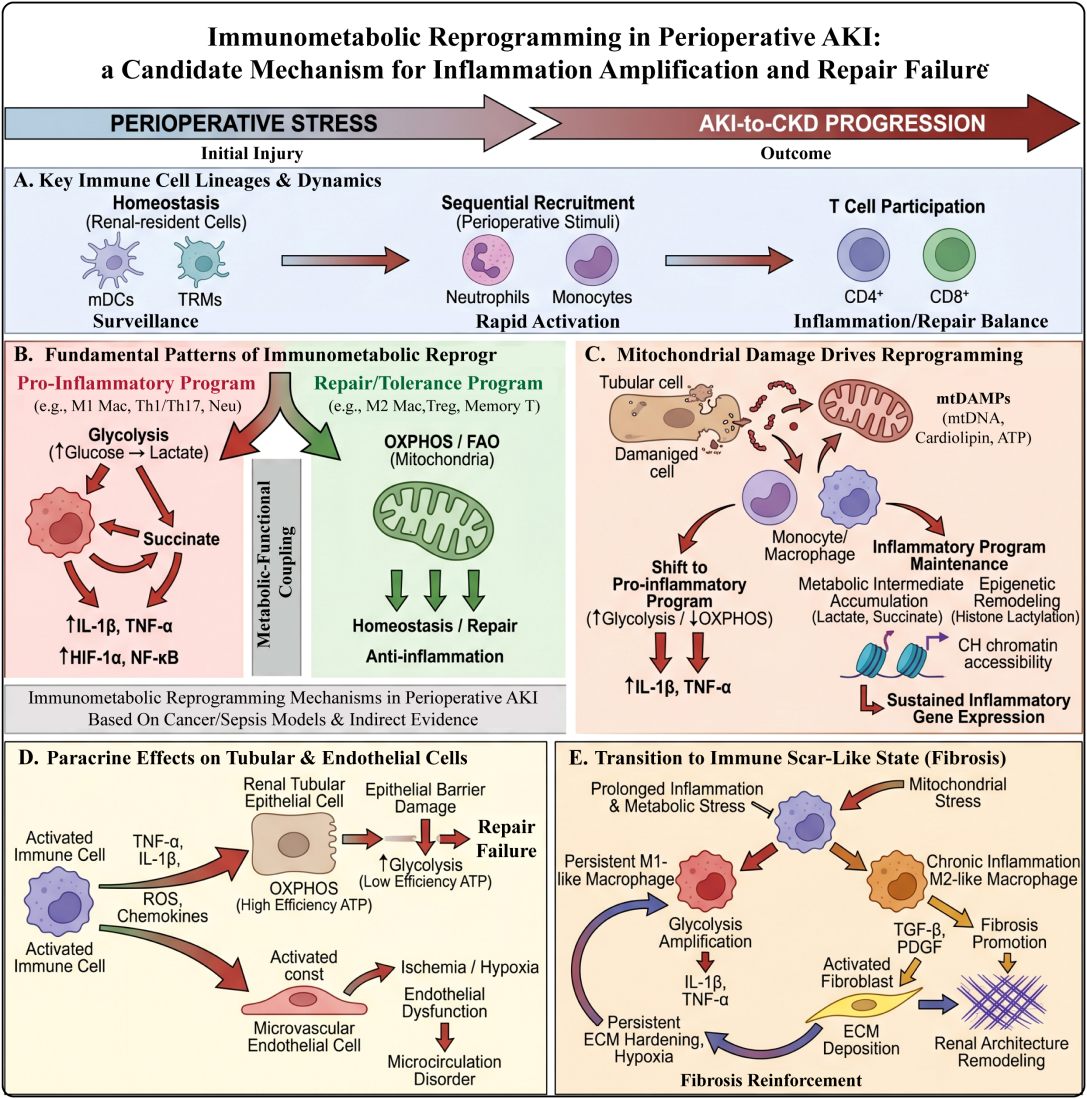
Immunometabolic reprogramming as a candidate mechanism driving inflammation amplification, repair failure, and AKI-to-CKD progression in perioperative AKI. **(A)** Key immune cell lineages and dynamics: Perioperative stress disrupts renal immune homeostasis and induces sequential recruitment and activation of neutrophils, monocytes/macrophages, and T cells. **(B)** Fundamental patterns of immunometabolic reprogramming: Immune cells shift between a pro-inflammatory glycolytic program and a repair/tolerance-associated oxidative phosphorylation and fatty acid oxidation program. **(C)** Mitochondrial damage-driven reprogramming: Injured tubular cells release mitochondrial damage-associated molecular patterns, including mtDNA, cardiolipin, and ATP, thereby promoting pro-inflammatory monocyte/macrophage reprogramming and sustained inflammatory gene expression. **(D)** Paracrine effects on tubular and endothelial cells: Activated immune cells release TNF-α, IL-1β, ROS, and chemokines, leading to tubular epithelial barrier damage, microvascular endothelial dysfunction, hypoxia, and repair failure. **(E)** Transition to an immune scar-like fibrotic state: Persistent inflammation and metabolic stress promote M1-like macrophage persistence, M2-like macrophage-associated profibrotic signaling, fibroblast activation, extracellular matrix deposition, and renal architectural remodeling, contributing to AKI-to-CKD progression.

If acute inflammation is not promptly resolved, immunometabolic imbalance may drive the kidney from acute injury toward chronic fibrosis, characterized by a long-term dominance of pro-inflammatory immune phenotypes and suppression of repair and tolerance programs (103, 104). In this process, some macrophages remain in a high-glycolysis, inflammation-amplifying M1-like state, continuously releasing cytokines such as IL-1β and TNF-α; others, under chronic inflammation and metabolic stress, acquire an M2-like pro-fibrotic phenotype, persistently secreting factors like TGF-β and PDGF (105, 106). Meanwhile, TGF-β can activate fibroblasts and promote excessive ECM deposition, driving renal structural remodeling (107). Local ECM stiffening, perfusion abnormalities, hypoxia, and mitochondrial stress can form a self-reinforcing cycle, perpetuating the inflammation-fibrosis process (108). Combined with the mitochondrial metabolic memory described in Section 3.5, AKI-to-CKD progression can be understood as the superposition of multiple “memory effects” at the epithelial, immune, and stromal levels. Targeting immunometabolic imbalance, macrophage pro-fibrotic polarization, or the TGF-β signaling axis may represent potential directions for alleviating the immune scar-like state, though their translational value in perioperative AKI requires further validation (**Figure 4**).

## 5 Integration of the pathological triangle model: a positive feedback network of renal microcirculatory hypoxia–mitochondrial damage–immunometabolic reprogramming

Existing AKI frameworks have explained the occurrence and progression of AKI from perspectives such as hemodynamic injury, renal tubular stress, mitochondrial dysfunction, and inflammation–metabolism crosstalk. These frameworks provide an important foundation for understanding AKI, but in perioperative settings, there is still limited emphasis on the correspondence among time window changes, dominant imbalance dimensions, and stratified interventions. The pathological triangle model proposed in this article does not treat microcirculation, mitochondria, and immune-inflammatory mechanisms as isolated new mechanisms but organizes them into a perioperative dynamic sequence: renal microcirculatory hypoxia as an early trigger, mitochondrial damage as an intracellular amplifier, and immunometabolic reprogramming as a sustaining module for persistent inflammation and failed repair. Thus, this model can propose testable hypotheses, including candidate biomarkers at different time windows, different dominant imbalance phenotypes, and phenotype-enriched intervention strategies.

### 5.1 Temporal sequence of the three dimensions of the pathological triangle model: dominant pathological events in different perioperative time windows

The occurrence of perioperative AKI is not a “discrete event” of a single mechanism at a single time point but rather a dynamic evolution process in which the three core dimensions of the proposed model-renal microcirculatory hypoxia, mitochondrial damage, and immunometabolic reprogramming—become relatively dominant and gradually coupled across different time windows (109, 110). It is important to emphasize that this temporal sequence is more suitable as a conceptual dominant pattern rather than a uniform timeline strictly followed by all patients; in contexts of high inflammatory baselines or severe ischemia–reperfusion, the three dimensions of the pathological triangle model can exhibit significant overlap or even synchronous activation.

In the very early stage during and within hours after surgery, the most prominent dimension is renal microcirculatory hypoxia, the upstream trigger in the pathological triangle model. Hemodynamic fluctuations, changes in volume status, the effects of anesthetic drugs on renal vascular autoregulation, and surgical trauma itself can cause rapid changes in renal blood flow perfusion and oxygen supply (111). Within this time window, the cortical–medullary oxygen gradient is disrupted, with the medullary region, especially the outer medulla, first progressing to pathological hypoxia. OXPHOS in renal tubular epithelial cells (TECs) is inhibited, and energy reserves are rapidly depleted (112). Although endothelial activation and coagulation–inflammatory responses have already begun, immune cell infiltration and immunometabolic reprogramming mostly remain in an early preparatory state. Therefore, this stage can be regarded as the initiation period where the microcirculation dimension is relatively dominant (113).

Entering the early stage approximately 6–72 hours postoperatively, with the occurrence of reperfusion, the pathological focus gradually shifts to the mitochondrial crisis in the “energy dimension” of the pathological triangle model (114). Reperfusion-related burst of reactive oxygen species (ROS), calcium overload, and mPTP opening collectively lead to ATP synthesis impairment, membrane potential collapse, and mitochondrial quality control imbalance, triggering various forms of cell death (115, 116). Alongside mitochondrial damage, mtDAMPs such as mtDNA, cardiolipin, and extracellular ATP are released in large quantities, providing “fuel” for subsequent immunometabolic reprogramming (117). Simultaneously, immune cells such as macrophages and neutrophils infiltrate renal tissue, gradually shifting toward a pro-inflammatory metabolic program characterized by high glycolysis, marking a significant amplification of the linkage between the energy and inflammation dimensions in the pathological triangle model (118). In the intermediate to late stage from several days to weeks postoperatively, immunometabolic reprogramming and fibrotic tendencies gradually become key determinants of outcomes (119). If the inflammation–metabolism network triggered by earlier microcirculatory hypoxia and mitochondrial damage fails to be effectively “shut down,” it often manifests as prolonged dominance of pro-inflammatory immune phenotypes and sustained suppression of reparative and tolerogenic programs (120). Renal tubular epithelial cells and infiltrating immune cells remain in a metabolic state of “high glycolysis–high ROS–low OXPHOS,” characterized by the accumulation of metabolites such as lactate and lipid peroxidation products; coupled with persistent mtDAMP signaling, this state promotes the gradual establishment of fibrosis (121, 122). Therefore, in this stage, the immunometabolic dimension becomes more dominant and, together with the first two dimensions, forms a positive feedback loop, determining whether the kidney enters effective repair or progresses toward AKI-CKD transition. This dynamic evolution provides the basis for the subsequent formation of a self-reinforcing feedback network.

### 5.2 Key signaling pathways and self-reinforcing feedback networks

In the pathological triangle model of “renal microcirculatory hypoxia–mitochondrial damage–immunometabolic reprogramming,” several key signaling axes collectively connect perfusion/oxygenation abnormalities, mitochondrial energy crisis, and immune network remodeling, forming mutually reinforcing pathological networks. This section focuses on how these molecular nodes interconnect the components of the pathological triangle model and drive the evolution of perioperative AKI from acute injury to persistent inflammation and impaired repair. First, the hypoxia-HIF axis can be regarded as the “molecular translator” converting microcirculatory hypoxia into metabolic and inflammatory remodeling (123). Perioperative hypoperfusion, blood loss, hemodilution, or blood flow redistribution can lead to decreased renal medullary oxygenation, causing HIF-1α to shift from rapid degradation to stable accumulation, promoting a cellular shift from OXPHOS to glycolysis, and inducing the expression of pro-inflammatory and adaptive response molecules (124). Therefore, the core significance of the HIF axis lies in translating local oxygenation abnormalities into changes in energy metabolism and inflammatory responses, rather than merely serving as a hypoxia marker. Second, pathways related to mtROS and mtDAMPs constitute the “bridge amplifier” between mitochondrial damage and innate immune activation. mtROS, mtDNA, cardiolipin, and extracellular ATP released after mitochondrial damage can trigger innate immune responses through pathways such as cGAS–STING, TLR9, P2X7, and NLRP3 (125). This process rapidly expands the energy crisis originally confined within renal tubular epithelial cells or endothelial cells into an inflammatory amplification loop involving macrophages, dendritic cells, and other immune cells. Thus, a direct pathological connection is formed between the energy and inflammation dimensions of the pathological triangle model, explaining why mitochondrial damage can continuously amplify local inflammation. Third, the AMPK and Sirtuin families can be regarded as “metabolic dimmers” in the pathological triangle model (126). AMPK promotes mitophagy and fatty acid oxidation during energy deficiency and limits excessive glycolysis and inflammatory drive; Sirt1, Sirt3, among others, regulate the activities of HIF-1α, NF-κB, and various metabolic enzymes through NAD^+^-dependent deacetylation, participating in hypoxia adaptation, antioxidant defense, and inflammation suppression (127–129). Therefore, AMPK/Sirtuins are not single protective pathways but important systemic hubs that simultaneously sense microcirculatory oxygen supply, mitochondrial energy status, and immunometabolic shifts.

Based on the above signaling axes, perioperative AKI can form three representative self-reinforcing feedback loops. The first is the “microcirculatory hypoxia-mitochondrial ROS-endothelial damage/microthrombosis” loop. Hypotension, blood loss, sepsis-related blood flow redistribution, or cardiopulmonary bypass can lead to insufficient renal microcirculatory perfusion, causing the medulla and renal tubular regions to become hypoxic (130, 131). Hypoxia inhibits ETC activity and induces increased mtROS, which further damages mitochondria and endothelial cells, promoting vasoconstriction, adhesion molecule expression, and microthrombosis, ultimately exacerbating perfusion heterogeneity and the “no-reflow” phenomenon (132, 133). As a result, initial hypoxia is amplified into more severe tissue hypoxia and an energy crisis. The second is the “mitochondrial DAMPs-immune activation–inflammatory factors–secondary mitochondrial damage” loop. When mitochondrial damage exceeds quality control capacity, mtDAMPs such as mtDNA, cardiolipin, and ATP can leak into the cytosol or extracellular space, activating macrophages and dendritic cells and initiating pathways such as cGAS-STING and the NLRP3 inflammasome (134, 135). Subsequently released inflammatory factors and ROS/RNS in turn damage renal tubular epithelial cells and endothelial cells, inducing more mitochondrial rupture and mtDAMP release (136). This loop transforms focal mitochondrial crisis into persistent inflammatory amplification. The third is the “pro-inflammatory immunometabolism-repair inhibition-structural remodeling–persistent hypoxia” loop. In the intermediate to late postoperative stage, if immunometabolism remains skewed toward a pro-inflammatory state for an extended period, M1 macrophages and effector T cells can sustain high glycolysis and pro-inflammatory secretion, while the OXPHOS/fatty acid oxidation programs of M2 macrophages and Tregs are suppressed, leading to insufficient pro-repair signals (137, 138). Simultaneously, renal tubular epithelial cells persistently shift from efficient OXPHOS to inefficient glycolysis, reducing regenerative capacity and promoting fibroblast activation, extracellular matrix (ECM) deposition, capillary rarefaction, and interstitial fibrosis. Structural remodeling further exacerbates local hypoxia, maintaining the pro-inflammatory/pro-fibrotic state (139). Therefore, these signaling nodes provide the molecular basis for the mutual coupling of the three dimensions in the pathological triangle model.

### 5.3 Hypothesis of “imbalance patterns” for different clinical phenotypes of AKI in the triangle model

**Figure 5:**
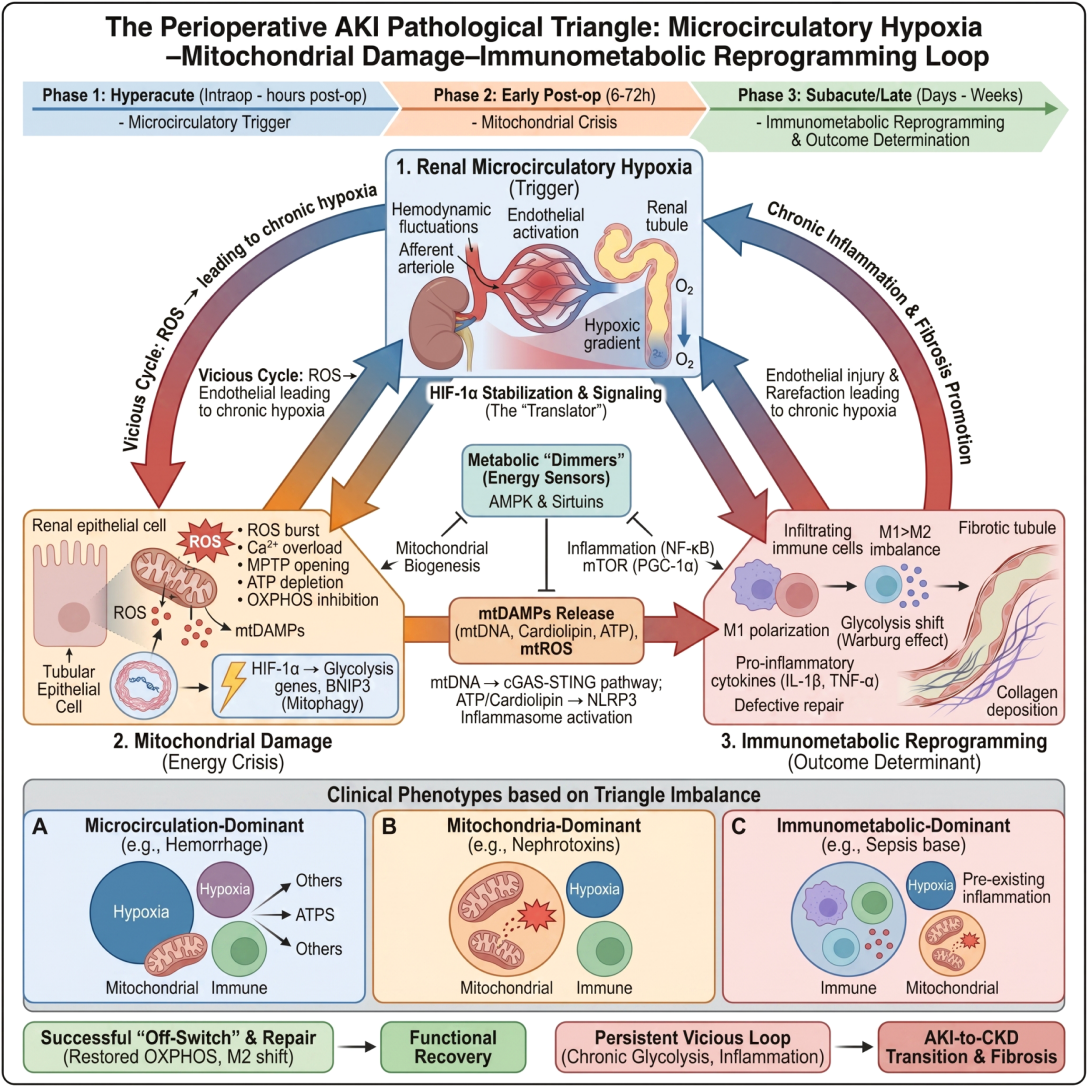
The perioperative AKI pathological triangle: a microcirculatory hypoxia–mitochondrial damage–immunometabolic reprogramming loop. Perioperative AKI may be initiated by renal microcirculatory disturbance, tubular hypoxia, and endothelial activation, with HIF-1α stabilization acting as a key translator between hypoxic stress, mitochondrial dysfunction, and immune-metabolic responses. During the mitochondrial injury phase, ROS burst, Ca^2+^ overload, mPTP opening, ATP depletion, and OXPHOS inhibition promote the release of mtDAMPs, including mtDNA, cardiolipin, ATP, and mtROS, thereby activating cGAS–STING and NLRP3 inflammasome signaling. Subsequent immune-cell infiltration is accompanied by glycolytic reprogramming, M1 polarization, and pro-inflammatory cytokine production, leading to defective repair, collagen deposition, and fibrosis. Based on the dominant component of triangle imbalance, perioperative AKI may present as **(A)** microcirculation-dominant; **(B)** mitochondria-dominant; or **(C)** immunometabolic-dominant phenotypes. Successful resolution of the loop supports functional recovery, whereas persistent vicious cycling promotes AKI-to-CKD transition and renal fibrosis.

AKI is not a homogeneous syndrome; there is significant heterogeneity in its clinical manifestations, pathological mechanisms, and outcomes (140). Under the pathological triangle model of “renal microcirculatory hypoxia–mitochondrial damage–immunometabolic reprogramming,” AKI in different clinical contexts can be understood as candidate phenotypes with varying degrees of imbalance and dominant sequences among the three dimensions of this framework: some are dominated by microcirculatory dysfunction, others centered on endogenous mitochondrial crisis, and still others present with obvious systemic immune–metabolic baseline abnormalities preoperatively (141–143). It is important to emphasize that the “microcirculation-dominant,” “mitochondria-dominant,” and “immunometabolism-dominant” types proposed in this article are currently hypothetical clinical phenotypes rather than validated diagnostic classifications. Their boundaries, diagnostic criteria, and biomarker cutoffs have not been confirmed in prospective cohorts or randomized clinical trials. Therefore, the main translational value of the pathological triangle model lies not in renaming existing mechanisms but in proposing stratified hypotheses that can be prospectively validated: different patients may exhibit imbalance patterns dominated by microcirculation, mitochondria, or immunometabolism, which can be tentatively identified using hemodynamic readings, renal resistive index (RRI), urinary mtDNA, NGAL, KIM-1, inflammatory factors, and immunometabolism-related markers. This hypothesis of “dominant imbalance patterns in the pathological triangle model” helps transcend the reliance on serum creatinine (Scr) and urine output alone, explain the heterogeneity of AKI at the pathophysiological level, and provide a conceptual foundation for stratified diagnosis and targeted intervention. To enhance clinical translatability, this article further proposes candidate clinical readings and exploratory criteria for each type of imbalance pattern for future cohort studies, mechanism validation, and phenotype-enriched trials; however, these proposals should not be construed as current clinical diagnostic standards (**Figure 5**).

#### 5.3.1 “Microcirculation-dominant type”: hemodynamically driven AKI

The “microcirculation-dominant type” AKI is commonly seen in perioperative situations with massive hemorrhage, severe shock, or significant hemodynamic fluctuations. In this context, decreased effective circulating blood volume and reduced perfusion pressure are the main upstream drivers, with redistribution of renal cortical-medullary perfusion and absolute medullary anoxia predominating in the microcirculation dimension of the pathological triangle model (144). Clinically, such patients often present with early oliguria or anuria, and AKI is closely related to hypotension, blood loss, and the need for vasoactive drug support, and the condition is typically responsive to volume resuscitation and individualized blood pressure management (145, 146). Candidate diagnostic features may include: intraoperative mean arterial pressure (MAP) below the individualized autoregulation threshold or prolonged hypotension, such as MAP <65 mmHg lasting >30 min; significant blood loss or negative fluid balance; early oliguria; elevated RRI; BOLD-MRI, near-infrared spectroscopy, or other tissue oxygenation assessments indicating renal medullary anoxia; and a lack of significant elevation in markers of mitochondrial injury, such as U-mtDNA not significantly increased. If microcirculatory hypoxia can be promptly corrected during or early after surgery, mitochondrial injury and immune activation may be relatively reversible; conversely, persistent microcirculatory failure will quickly involve the mitochondrial injury and immune-metabolic dimensions of the pathological triangle model (147, 148). Therefore, this candidate phenotype represents an ideal target for clinical investigations into individualized blood pressure management, microcirculation monitoring, volume optimization, and tissue oxygenation-targeted therapy.

#### 5.3.2 “Mitochondria-dominant type”: intracellular energy crisis-driven AKI

The “mitochondria-dominant type” AKI is characterized by a specific and concentrated direct hit to mitochondria, occupying a dominant position in the pathological triangle (149). Typical scenarios include cardiac surgery with prolonged cardiopulmonary bypass, and exposure to nephrotoxic drugs such as cisplatin or aminoglycosides. In these patients, initial hemodynamic disturbances may not be significant or have been quickly corrected, but factors such as drug accumulation in renal tubular cells and reperfusion stress lead to particularly prominent ETC inhibition, mtROS burst, and mPTP opening, with ATP depletion and cell death becoming decisive events (150, 151). Clinically, these patients may present with asynchronous elevation of Scr and changes in urine output, insignificant conventional imaging perfusion changes, and relatively slow recovery of renal function (152); candidate diagnostic features may include: clear exposure to mitochondrial toxicity or prolonged ischemia-reperfusion background; elevated U-mtDNA, urine NGAL, KIM-1, or mitochondria-related metabolites; abnormal lactate/pyruvate ratio, TCA cycle intermediates, or acylcarnitine profiles; and absence of persistent severe hypotension or significant RRI elevation. In the pathological triangle model, these patients primarily exhibit dominance in the mitochondrial injury dimension, suggesting that mitochondrial protection, metabolic support, and reduction of reperfusion injury may be more appropriate intervention focuses (153, 154). However, there are currently no clinically validated U-mtDNA or metabolomic cutoffs to define this phenotype, and related indicators should mainly serve as exploratory stratification tools.

#### 5.3.3 “Inflammation-dominant type”: systemic inflammation-metabolic high-risk background-driven AKI

The “inflammation-dominant type” AKI mostly occurs in patients with pre-existing significant systemic inflammation or metabolic disorders, such as those with Diabetes Mellitus, obesity/metabolic syndrome, autoimmune diseases, or those undergoing major surgery, severe infection, and sepsis treatment (155, 156). In this population, the perioperative insult is superimposed on an existing immune-metabolic “high-risk baseline,” predisposing innate immune cells toward a high-glycolytic, pro-inflammatory polarization state, resulting in the excessive release of cytokines and chemokines, leading to renal parenchymal injury and microenvironment collapse (157). Such patients often present with a relatively late onset of AKI and a delayed yet persistent disease course, frequently complicated by multi-organ dysfunction. Candidate diagnostic features may include: preoperative background of Diabetes Mellitus, obesity/metabolic syndrome, chronic inflammatory diseases, or infection; elevated hs-CRP, IL-6, TNF-α, SII, or NLR; abnormal urine cell factors, complement activation markers, or immune-related molecular subphenotypes; and the presence of a persistent inflammatory response and multi-organ involvement. In the pathological triangle model, these patients primarily exhibit an immune-metabolic dimension that is in a “pre-activated” state before surgery, and perioperative intervention should not only focus on perfusion optimization and mitochondrial function but also emphasize the assessment and regulation of the immune-metabolic baseline (158, 159). This phenotype can serve as a foundation for phenotype-enriched studies on anti-inflammatory, immune-metabolic modulation, complement inhibition, or infection control strategies, but its clinical identification still requires standardized inflammatory markers and molecular subtype validation (**Table 1**).

**Table 1:** Proposed “imbalance phenotypes” and exploratory diagnostic criteria in the perioperative AKI triangle model.

Candidate phenotype	Primary driver	Candidate clinical scenario	Candidate diagnostic indicators	Potential intervention direction	Current evidence status
Microcirculation-Dominant	Renal hypoperfusion, Cortical- medullary oxygenation imbalance.	Massive hemorrhage, Shock, Prolonged hypotension, Vasoactive drug dependence.	MAP <65 mmHg for >30 min or below individualized autoregulation threshold; Significant blood loss/hypovolemia; Early oliguria; Elevated RRI; BOLD-MRI findings or tissue oxygenation metrics indicative of medullary hypoxia; U-mtDNA not significantly elevated.	Individualized blood pressure management, Volume optimization, Microcirculation monitoring, Tissue oxygenation-guided therapy.	Hypothetical phenotype, lacks validated cutoffs.
Mitochondria- Dominant	Energy crisis in renal tubular cells, Release of mtROS and mtDAMPs	Prolonged cardiopulmonary bypass, Ischemia- reperfusion, Nephrotoxic drug exposure.	Elevated U-mtDNA; Elevated NGAL/KIM-1; Abnormalities in TCA cycle, acylcarnitines, or lactate/pyruvate ratio; Asynchronous rise in Scr and changes in urine output; No persistent severe hypotension or significant RRI elevation.	Mitochondrial protection, antioxidant therapy, metabolic support, and reperfusion injury reduction.	Exploratory stratification, no unified biomarker cutoff.
Immune- Metabolic Dominantmolecular subtyping and prospective validation	Preoperative inflammatory/metabolic high-risk baseline and postoperative immune amplification	Diabetes Mellitus, Obesity/MetS, Autoimmune diseases, Infection, Sepsis, Major surgery	Elevated hs-CRP, IL-6, TNF-α, SII/NLR; Abnormal urinary cytokines or complement activation markers; Multiple organ dysfunction; Late-onset and persistent AKI.	Anti-inflammatory therapy, Immune-metabolic regulation, Complement/cell factor targeting, Infection control.	Candidate phenotype, requires.

## 6 Diagnosis and stratification strategy based on the pathological triangle model

### 6.1 Interpretation of the limitations of traditional AKI diagnostic indicators (Scr, urine output) in the triangle model

Scr and urine output remain the foundational indicators for KDIGO diagnosis of AKI. However, under the “renal microcirculatory hypoxia–mitochondrial injury–immune metabolic reprogramming” pathological triangle model, their lag and non-specificity become more pronounced (160). Scr essentially reflects the glomerular filtration rate (GFR), with a time delay in its changes, and is influenced by factors such as muscle mass, age, sex, diet, and medications. Therefore, it may underestimate early or mild renal injury (161, 162). From the perspective of the pathological triangle model, Scr elevation often occurs at a stage where microcirculatory disturbances, mitochondrial dysfunction, and immune metabolic disorders have already mutually amplified each other, making it closer to a “final reading” and difficult to reveal early mechanistic injury (163).

A decrease in urine output also lacks mechanistic specificity. In microcirculation-dominant AKI, it may primarily reflect decreased perfusion pressure and hemodynamic adaptation; in mitochondria-dominant AKI, it may be associated with tubular energy crisis, necrosis and shedding, and luminal obstruction; in immune metabolism-dominant AKI, inflammation-mediated endothelial injury, microthrombi, and interstitial inflammation may also lead to decreased urine output (164, 165). Cardiac surgery studies suggest that a significantly higher proportion of patients are diagnosed with AKI based solely on urine output criteria than on Scr criteria, but some of these patients do not exhibit clear elevations in injury markers or adverse outcomes, suggesting potential “overclassification” by urine output criteria (166). Therefore, Scr and urine output are more suitable as basic identification tools for AKI but are inadequate for mechanistic subtyping. In other words, Scr and urine output have high clinical accessibility and standardization advantages, but their sensitivity for early injury is limited, and their specificity for microcirculation-, mitochondria-, or immune metabolism-dominant injury is insufficient.

### 6.2 Imaging and functional assessment related to renal microcirculation and tissue hypoxia

In the pathological triangle model, renal microcirculatory hypoxia is a relatively upstream triggering factor. Identifying the state of “normal macrovascular blood pressure but local renal hypoxia” during the perioperative period is an important prerequisite for early warning and stratified intervention (167). In terms of application maturity, renal Doppler and the renal resistive index (RRI/RI) are more established in clinical practice, while BOLD-MRI, NIRS, and CEUS remain largely confined to the research or early translational stage (168, 169). Renal Doppler ultrasound can indirectly reflect intrarenal vascular resistance and microcirculatory perfusion status through the RRI. An elevated RRI is associated with AKI occurrence and poor prognosis, serving as a bedside surrogate marker for impaired microcirculatory function and providing auxiliary information for fluid management and vasoactive drug adjustment (170, 171). However, the RRI is influenced by age, arterial stiffness, intra-abdominal pressure, right heart function, vascular tone, and operator factors, making its sensitivity and specificity unstable. Thus, it is more suitable as a risk-stratification aid rather than an independent diagnostic indicator. BOLD-MRI can non-invasively assess oxygen tension differences between the renal cortex and medulla, making it suitable for identifying the occult state of “normal macrovascular flow but medullary hypoxia”; NIRS holds promise for continuous perioperative monitoring of renal oxygenation, but direct clinical evidence remains limited (172–174). CEUS, BOLD-MRI, and NIRS have high value in mechanistic studies, but due to limitations in device accessibility, perioperative operating conditions, standardized thresholds, and outcome validation, they are currently not suitable as routine bedside stratification tools.

### 6.3 Mitochondrial function and injury markers

The mitochondrial injury dimension of perioperative AKI primarily involves renal tubular mitochondrial dysfunction and the release of mtDAMPs. Currently, urinary or circulating mtDNA, NGAL, KIM-1, and several targeted metabolites have high translational potential, but most still lack unified cut-off points and prospective validation (175, 176). In terms of clinical readiness, NGAL and KIM-1 have a more robust research foundation, with good sensitivity for early injury, but their specificity is limited and may be affected by inflammation, infection, underlying CKD, surgical type, and extrarenal factors. U-mtDNA more accurately reflects local tubular mitochondrial injury, while cf-mtDNA encapsulates data regarding both local and systemic inflammation, potentially reflecting the connection between mitochondrial crisis and inflammatory amplification (177, 178). However, U-mtDNA/cf-mtDNA currently lacks standardized detection protocols, unified measurement units, and clinical cut-off points, making them more suitable as exploratory enrichment indicators for mitochondria-dominant AKI rather than bedside decision-making indicators. Additionally, targeted metabolites such as succinate and acylcarnitines can provide a “functional fingerprint” of mitochondrial dysfunction, helping to identify different patterns of energy imbalance (179). In comparison, TFAM, cytochrome c, and other mitochondria-related proteins have mechanistic rationale but limited direct clinical evidence in perioperative AKI. Therefore, these indicators are more suitable as exploratory research tools and should not be described as stratification markers nearing routine application.

### 6.4 Immune and metabolic biomarkers

In the pathological triangle model, the immune metabolism dimension primarily reflects inflammatory activation, immune cell metabolic reprogramming, and their impact on the local renal microenvironment (180). Currently, more practically relevant indicators include conventional inflammatory markers and urinary cytokine profiles; immune cell metabolic phenotypes and multi-omics clustering remain primarily future research directions. Systemic inflammatory scores, such as the systemic immune-inflammation index (SII) and the neutrophil-to-lymphocyte ratio (NLR), can reflect systemic inflammatory tone and serve as simple tools for identifying high-risk immune metabolic backgrounds (181–183). These indicators are readily available and cost-effective, suitable for perioperative risk screening, but their low specificity hinders the differentiation of infection, surgical trauma, underlying inflammatory states, and AKI-related immune activation. Urinary cytokine profiles are closer to the renal local microenvironment; for example, elevations in IL-6, IL-8, and TNF-α can indicate increased local inflammation and microenvironmental disruption (184). However, the detection platforms, thresholds, sampling times, and prognostic value of urinary cytokine panels have not been standardized, currently limiting their utility to research stratification tools for immune metabolism-dominant AKI. In contrast, assessing immune cell glycolysis/OXPHOS status via flow cytometry combined with Seahorse, as well as identifying immune–metabolic subphenotypes through metabolomics, lipidomics, and single-cell omics, while having high mechanistic value, are still in the discovery and validation phase and are far from routine perioperative application (185, 186).

### 6.5 A stratified biomarker framework based on the pathological triangle model

To avoid simply listing various tools in parallel, this article proposes classifying perioperative AKI-related indicators into three tiers based on clinical readiness. The first tier is the currently feasible clinical foundation layer, primarily including Scr, urine output, baseline eGFR, blood pressure, fluid balance, lactate, hemoglobin, intraoperative hypotension duration, blood loss, extracorporeal circulation time, nephrotoxic drug exposure, conventional inflammatory markers, and, when necessary, renal Doppler/RRI. This layer has high accessibility and is suitable for routine risk identification and dynamic monitoring, but its mechanistic specificity is limited. The second tier is the near-to-medium-term translational layer, including NGAL, KIM-1, U-mtDNA/cf-mtDNA, urinary cytokine panels, NIRS, CEUS, BOLD-MRI, and targeted metabolites. These tools can enhance early injury identification or mechanistic stratification capacity but still require standardized detection protocols, sensitivity/specificity evaluation, standardized cutoff values, and prospective outcome validation. The third tier is the mechanistic exploration layer, including immune cell metabolic phenotypes, mitochondrial function assays, single-cell omics, metabolomics, lipidomics, multi-omics clustering, and machine learning integration models. These tools are suitable for mechanism discovery, endotype exploration, and phenotype enrichment trial design but are not yet suitable for routine bedside decision support. In the clinical workflow, a more practical path is not to test all biomarkers simultaneously but to adopt a stepwise strategy: first, use KDIGO indicators and routine perioperative risk factors for basic identification; second, in high-risk or early suspected AKI patients, add bedside readings with higher accessibility, such as RRI, lactate, fluid balance, inflammatory markers, and some early renal injury markers; finally, in research settings, further integrate U-mtDNA, urinary cytokines, BOLD-MRI/CEUS, metabolomics, and multi-omics tools to validate candidate phenotypes of microcirculation-dominant, mitochondria-dominant, and immune metabolism-dominant AKI.

### 6.6 Minimum biomarker panel for prospective perioperative trials

For prospective perioperative trials in adults or children, the minimum biomarker panel should prioritize clinical accessibility, feasibility of repeated sampling, mechanistic relevance, and cross-center standardization, instead of initial reliance on complex multi-omics platforms. Therefore, this article recommends a pragmatic minimum indicator set: all patients should have KDIGO indicators and routine perioperative risk variables recorded; on this basis, RRI/lactate, NGAL/KIM-1/U-mtDNA, and hs-CRP/IL-6/NLR or SII can be respectively combined for exploratory identification of candidate phenotypes of microcirculatory hypoxia-dominant, mitochondrial injury-dominant, and inflammation-dominant AKI. For adult trials, the above minimum combination can be jointly modeled with baseline variables such as CKD, diabetes, obesity, heart failure, infection status, and surgical risk grade. For pediatric trials, special attention should be paid to age-related reference ranges, body surface area correction, blood volume limitations, and developmental variations in renal function and immune response maturity. BOLD-MRI, CEUS, NIRS, metabolomics, single-cell omics, and immune cell metabolic function assays can serve as extended research tools and should not be mandatory items in the minimum trial panel (**Table 2**).

**Table 2:** Perioperative AKI diagnosis and stratification tools and evidence levels based on the pathological triangle model.

Category	Representative indicators/methods	Corresponding pathological dimension	Primary stratification positioning	Evidence level/clinical maturity	Reference
Traditional Diagnostic Indicators	Scr, Urine Output	Integrated terminal functional reading	Used for basic AKI identification and KDIGO grading, not suitable for mechanistic subtyping.	Clinical routine	(161–163)
Microcirculation Assessment	Renal Doppler, RI/RRI	Renal microcirculatory hypoxia	Assists in identifying microcirculatory perfusion abnormalities and supports bedside risk assessment.	Clinical adjunct/ Partial clinical routine	(168–171)
Renal Oxygenation Imaging/Monitoring	BOLD-MRI, NIRS, CEUS	Renal microcirculatory hypoxia	Identifies occult renal hypoxia, supports microcirculatory and tissue oxygenation stratification.	Research tool/Early translation	(172–174)
Mitochondrial Injury Markers	NGAL, KIM-1, U-mtDNA, cf-mtDNA	Mitochondrial injury	Explores early renal tubular injury, mitochondrial injury-dominant AKI, and mtDAMPs release status.	Research tool/ Partial early translation	(177, 178)
Mitochondrial Functional	Metabolites Succinate, acylcarnitines, etc. (targeted metabolites)	Mitochondrial injury	Reflects energy imbalance patterns, supports functional mechanistic stratification.	Research tool/ Mechanism exploration.	(179)
Conventional Inflammatory Index	SII, NLR, hs-CRP, etc.	Immune metabolic reprogramming/ Inflammatory burden.	Identifies high inflammatory burden background and inflammation-dominant risk.	Clinical routine/ Clinical adjunct, but with limited specificity.	(181–183)
Local Inflammatory Markers	Urine cytokine panel, e.g., IL-6, IL-8, TNF-α, etc.	Immune metabolic reprogramming	Reflects the local renal inflammatory microenvironment and supports stratification of inflammation-dominant candidate phenotypes.	Research tool	(184)
Frontier Mechanistic Tools	Immune cell metabolic phenotype, multi-omics clustering, machine learning integration.	Comprehensive dimensions of the pathological triangle model.	Used for endotype exploration, precision stratification, and prognostic prediction model construction.	Research tool/Hypothesis generation.	(185, 186)
Minimum Prospective Trial Combination	KDIGO indicators, routine perioperative risk variables, RRI/lactate, NGAL/KIM-1/U-mtDNA, hs-CRP/IL-6/NLR or SII	Comprehensive assessment of three pathological dimensions	Used for exploratory phenotype identification in adult or pediatric prospective trials.	Research stratification combination, not yet a clinical diagnostic standard.	–

### 6.7 Currently available content and future research directions

Currently, the pathophysiological triangle model should not be directly used as an independent diagnostic tool. Its current operational value primarily lies in helping clinicians reconceptualize the sources of perioperative AKI risk and, based on the existing KDIGO monitoring framework, enhance awareness of different injury origins. At this stage, the content applicable to clinical practice includes: preoperative baseline eGFR and Scr assessment, routine risk stratification based on age, CKD, diabetes mellitus, heart failure, anemia, infection, and major surgery type; intraoperative monitoring of hypotension duration, blood loss, hemodilution, and extracorporeal circulation time; and postoperative dynamic observation of Scr and urine output. Treatment should remain grounded in the KDIGO bundle, encompassing the avoidance of nephrotoxic agents, optimization of volume and perfusion, control of infection and blood glucose, and maintenance of electrolyte and acid-base balance. Conversely, U-mtDNA, RRI, BOLD-MRI, CEUS, mitochondrial metabolite profiles, urinary cytokines, complement activation markers, and immunometabolic characteristics remain research or exploratory tools. They can be used for prospective cohort enrichment, mechanism validation, and phenotype stratification studies, but before unified cutoffs, standardized detection processes, and prospective outcome validation are established, they should not be directly applied to routine bedside decision-making. Similarly, microcirculation-dominant, mitochondria-dominant, and immunometabolism-dominant AKI are currently candidate phenotypes rather than validated clinical diagnostic categories. Future research should focus on verifying whether these candidate biomarkers and phenotypes can reliably predict AKI occurrence, recovery trajectory, AKI-CKD transition risk, and response to specific intervention strategies.

## 7 Treatment and intervention targeting the pathological triangle model: from “single-point correction” to “multi-angle linkage”

### 7.1 Microcirculation and oxygen supply optimization strategies

The prevention and treatment of perioperative AKI remain based on maintaining effective perfusion, oxygen delivery, and renal microcirculatory stability (187, 188). In shock, ischemia-reperfusion, or complex perioperative stress situations, even when macro-hemodynamic parameters such as mean arterial pressure are within an acceptable range, renal medullary hypoperfusion and occult hypoxia may still exist (189). Therefore, macro-hemodynamic optimization is a necessary condition but does not equate to complete recovery of renal microcirculation and medullary oxygenation. Individualized goal-directed hemodynamic management, moderate fluid resuscitation, avoidance of severe hemodilution, and reduction of nephrotoxic exposure remain the most clinically actionable strategies (190–192). However, evidence for perioperative hemodynamic and oxygen supply optimization is not entirely consistent. Goal-directed fluid therapy, individualized MAP management, and oxygen supply optimization have shown benefits in some studies, but their renoprotective effects are inconsistent across different surgical types, baseline risks, anesthesia protocols, intervention timings, and endpoint definitions. Excessive fluid administration may exacerbate venous congestion, renal interstitial edema, and elevations in intrarenal pressure; over-reliance on vasoactive drugs may improve MAP but not necessarily synchronously improve renal microcirculatory perfusion and medullary oxygenation; and higher oxygen exposure may carry potential risks through oxidative stress. Therefore, the failure of some hemodynamic or oxygen supply regulation strategies to stably reduce AKI risk does not negate the importance of microcirculatory hypoxia but suggests that achieving a single macro-target is insufficient to represent the recovery of local renal oxygen supply. Overall, microcirculation and oxygen supply optimization are more suitable as foundational measures to block the initiation and amplification of injury. Future research should shift from “achieving blood pressure or oxygen supply targets” to individualized strategies guided by “microcirculation/tissue oxygenation monitoring, biomarkers, and dominant imbalance patterns.”

### 7.2 Targeted mitochondrial protection and repair

Mitochondrial protection is an important direction in translational research for perioperative AKI, primarily including inhibition of mtROS/mPTP, maintenance of mitochondrial quality control, and restoration of the NAD^+^–AMPK–Sirtuins axis (193, 194). First, inhibiting mtROS bursts and persistent mPTP opening aims to block the transition of mitochondria from reversible stress to irreversible collapse (195). Second, maintaining mitochondrial quality control includes promoting mitophagy, preserving biogenesis and dynamic balance to reduce the accumulation of damaged mitochondria and continuous leakage of mtDAMPs (196, 197). Third, restoring the NAD^+^–AMPK–Sirtuins axis, which represents energy sensing and metabolic homeostasis, aims to improve mitochondrial function, limit excessive mTOR activation, and to some extent correct the metabolic mismatch of “high glycolysis–low OXPHOS” (198). These strategies have strong mechanistic plausibility in cellular and animal models, theoretically reducing ATP depletion, mtDAMPs leakage, and secondary inflammatory amplification. However, it must be emphasized that mitochondrial-targeted therapy in perioperative AKI remains primarily in preclinical or early translational stages. Various antioxidants, mitochondrial protectants, or metabolic regulation strategies have shown benefits in animal models, but clinical trial results are unstable, often affected by dosing timing, dosage, patient heterogeneity, endpoint selection, and lack of phenotype enrichment design. Therefore, mitochondrial protection should not currently be described as a mature treatment regimen but is more suitable as a potential therapeutic avenue for mitochondria-dominant AKI.

### 7.3 Immunometabolic regulation and novel anti-inflammatory therapies

Immunometabolic regulation aims to block persistent inflammation and repair failure, rather than simply suppressing the inflammatory response (199, 200). Potential directions include modulation of the AMPK-mTOR axis, inhibition of the NLRP3 inflammasome, and activation of Sirtuins (201, 202). These strategies could theoretically reverse pro-inflammatory glycolytic shifts, reduce DAMP-driven inflammatory amplification, and promote the restoration of a reparative immune state. Among these, IL-6 blockade, such as tocilizumab, can serve as a testable candidate strategy in patients enriched for inflammatory markers. IL-6 is an important mediator in perioperative inflammatory response, endothelial activation, and immunometabolic shifts; its sustained elevation may be associated with AKI risk, systemic inflammatory burden, and multi-organ dysfunction (203). Therefore, in specific populations with significantly elevated IL-6 or persistent amplification of inflammatory response, IL-6 signaling blockade has certain mechanistic plausibility. However, tocilizumab lacks direct clinical validation in perioperative AKI, and its potential infection risk, immunosuppressive effects, and optimal dosing timing require careful evaluation. NLRP3 inflammasome inhibitors, such as MCC950, may also serve as candidate interventions for the mitochondrial damage-DAMP-inflammatory amplification axis. Given that mtDNA, oxidized cardiolipin, and extracellular ATP, along with other mtDAMPs, contribute to NLRP3 inflammasome activation, selective NLRP3 inhibition could theoretically attenuate IL-1β/IL-18 release and subsequent inflammatory cascade amplification (204). MCC950 has shown mechanistic potential in various preclinical models of inflammation and AKI but currently remains primarily in the experimental research stage and cannot be considered a mature treatment option for perioperative AKI. However, the clinical translation of anti-inflammatory and immunomodulatory strategies in AKI still faces significant challenges (205). Broad-spectrum anti-inflammatory or antioxidant interventions often fail to stably improve clinical outcomes, possibly due to the highly dynamic immune state in AKI, the dual role of inflammation in both injury and repair, narrow intervention time windows, and lack of mechanistic stratification of patients (206). Therefore, the immunometabolic targeting strategies described in this section, including IL-6 blockade, NLRP3 inhibition, AMPK-mTOR modulation, and Sirtuins activation, should all be understood as candidate research directions rather than treatment recommendations with established routine clinical application. Future validation should be prioritized in immunometabolic high-risk or inflammatory marker-enriched populations.

### 7.4 Multimodal comprehensive intervention pathway: time-windowed strategies across preoperative, intraoperative, and postoperative phases

The management of perioperative AKI should not be viewed as a single rescue at a specific time point but is better understood as a time-windowed, multimodal comprehensive intervention pathway spanning the preoperative, intraoperative, and postoperative phases (207, 208). Based on the pathological triangle model, the core logic of this pathway can be summarized as: reducing baseline vulnerability preoperatively, blocking the triggering of microcirculatory hypoxia intraoperatively, and preventing the solidification of mitochondrial crisis and continuation of immunometabolic bias postoperatively.

The key in the preoperative phase is to identify the “vulnerable kidney” and, to the greatest extent feasible, reduce the baseline imbalance across the three dimensions of the pathological triangle model. Advanced age, low prior eGFR, left ventricular dysfunction, a chronic inflammatory state, obesity, infection, and metabolic comorbidities may correspond to microcirculatory fragility, insufficient mitochondrial reserve, or chronic immunometabolic activation (209, 210). Therefore, the focus of preoperative multidisciplinary assessment is not merely on general risk stratification but also on minimizing the “potential imbalance baseline,” such as adjusting drugs affecting hemodynamic stability and correcting anemia, malnutrition, and electrolyte disturbances (211). The focus in the intraoperative phase is to prevent the microcirculatory hypoxia dimension of the pathological triangle model from being triggered first and to block its cascade extension to the mitochondrial injury dimension as much as possible. Goal-directed fluid therapy and the use of vasoactive drugs should still prioritize maintaining relatively stable perfusion and oxygen delivery, but this cannot be simply equated to elevating an isolated macro-parameter (212). The core of this phase is to minimize the depth and persistence of microcirculatory imbalance. The postoperative phase emphasizes early warning and phenotype-based intervention. If mechanistic markers such as U-mtDNA and urinary cytokine profiles can be further incorporated in the future, it may be possible to further refine “high-risk patients” into different dominant imbalance patterns. At this point, supportive treatment should still be based on KDIGO bundle management and ERAS pathways, but from the perspective of the pathological triangle model, the most important postoperative aspect is to prevent the solidification of early mitochondrial crisis and stop the continuous amplification of immunometabolic bias (147, 213). Therefore, the comprehensive management of perioperative AKI should be dynamically adjusted according to different time windows and dominant imbalance dimensions.

### 7.5 Bedside translation: stratified management pathways based on clinical scenarios

To enhance clinical interpretability, the pathological triangle model can be adapted into a supportive decision-making framework for common perioperative scenarios. These scenarios are not validated diagnostic algorithms but are intended to help clinicians identify major risk drivers and refine monitoring priorities based on the KDIGO bundle and routine perioperative management. First, patients with persistent intraoperative hypotension, significant blood loss, hypovolemia, or severe hemodilution may be more prone to microcirculation-dominant risk. Current actionable measures include correcting hypotension, optimizing volume and oxygen delivery, avoiding excessive hemodilution, dynamically monitoring urine output, lactate, Scr, fluid balance, and vasoactive drug requirements, and minimizing the administration of nephrotoxic agents (214). In the future, RRI, CEUS, BOLD-MRI, or NIRS could be further introduced to validate identification criteria for microcirculation-dominant AKI. Second, patients undergoing cardiac surgery, prolonged cardiopulmonary bypass, aortic clamping, severe ischemia-reperfusion, or exposure to nephrotoxic drugs may be more prone to mitochondria-dominant risk. The current focus is on mitigating the cumulative effects of ischemia-reperfusion and nephrotoxic exposure, closely monitoring Scr and urine output, and implementing KDIGO bundle management as early as possible. In the future, the value of U-mtDNA, NGAL, KIM-1, cf-mtDNA, and acylcarnitine profiles in identifying mitochondria-dominant AKI can be assessed. Third, patients with preoperative comorbidities such as diabetes, obesity/metabolic syndrome, infection, sepsis, chronic inflammation, or autoimmune diseases may be more prone to immunometabolic-dominant risk (215). Current actionable measures include controlling infection, optimizing blood glucose and metabolic status, assessing inflammatory burden, avoiding the superposition of hypoperfusion, nephrotoxic drugs, and inflammatory stimuli, and strengthening postoperative monitoring of Scr, urine output, inflammatory markers, and organ function. In the future, the value of IL-6, TNF-α, hs-CRP, SII, NLR, urinary cytokine profiles, and complement activation markers in phenotype enrichment can be validated.

### 7.6 From animal models to clinical trials: translational evidence gaps and clinical insights

Although the pathological triangle model provides an integrated framework for understanding perioperative AKI, most intervention evidence to date comes from single-hit animal models and non-perioperative disease contexts. First, the ecological validity of commonly used AKI animal models is limited, making it difficult to reflect real perioperative scenarios such as advanced age, multimorbidity, and repeated hits (216). Second, mismatches in dosing timing, dose, and target population are important reasons for clinical trial failures; the optimal windows for many interventions are inconsistent, and if this temporal difference is ignored, even reasonable targets may fail to show real benefits (217, 218). More fundamentally, AKI exhibits significant heterogeneity, and the dominant imbalance dimension in the pathological triangle model differs among patients; therefore, it is difficult to expect a single drug to uniformly address the full spectrum of AKI (219). A more feasible future translational pathway may be to first establish an operational phenotype classification system in prospective perioperative cohorts using markers related to microcirculation, mitochondria, and immunometabolism; then, based on this, conduct phenotype-enriched precision clinical trials, rather than enrolling all AKI patients into a single intervention framework. Overall, the pathological triangle model is currently more suitable as a “mechanism–classification–trial design” conceptual framework than as a directly applicable treatment guideline.

The clinical significance of the pathological triangle model does not lie in immediately replacing existing AKI diagnosis and treatment processes but in helping clinicians more systematically identify different risk factors of perioperative AKI. Normalization of mean arterial pressure does not equate to recovery of renal medullary oxygenation, and Scr and urine output, while necessary basic indicators, are delayed and non-specific. Therefore, early risk assessment of perioperative AKI should combine baseline renal function, duration of hypotension, blood loss, hemodilution, fluid balance, lactate levels, and exposure to nephrotoxic drugs. In the future, U-mtDNA, NGAL/KIM-1 may be used to explore mitochondria-dominant AKI, and hs-CRP, IL-6, NLR/SII, and urinary cytokine profiles may be used for research stratification of inflammation-dominant AKI, but these markers currently cannot replace clinical judgment. At this stage, management should still center on the KDIGO bundle and basic supportive therapy; the pathological triangle model is more suitable as a framework for risk identification and phenotype enrichment trial design, rather than an independent treatment algorithm (**Table 3**).

**Table 3:** Perioperative AKI treatment and intervention strategies based on the pathological triangle model, and evidence levels.

Intervention direction	Main strategies/representative content	Main action dimension	Evidence level/clinical maturity	Key issues/limitations	Translation positioning	References
Microcirculation and Oxygen Supply Optimization	Goal-directed hemodynamic management, moderate fluid resuscitation, vasoactive drug adjustment, maintaining oxygen-carrying capacity, avoiding severe hemodilution.	Renal microcirculatory hypoxia.	Clinical routine/Clinical foundation	Improvement in macro-hemodynamics does not guarantee restoration of local renal microcirculation and medullary oxygenation; excessive fluid resuscitation, overuse of pressors, or high oxygen exposure may all pose risks.	Currently the most actionable foundational intervention, suitable for early blocking of microcirculatory hypoxia triggers.	(189–192)
Targeted Mitochondrial Protection and Repair	Inhibiting mtROS/mPTP, maintaining mitochondrial quality control, restoring the NAD^+^–AMPK–Sirtuins axis	Mitochondrial damage	Preclinical/ Early translation	Most evidence comes from animal or cell models; clinical trial results are inconsistent; optimal timing, dose, and target population are not yet clear.	A candidate mechanism-guided strategy suitable for future phenotype-enriched trials focused on AKI dominated by mitochondrial damage.	(195–198)
Immunometabolic Regulation and Anti- inflammatory	Strategies AMPK–mTOR axis modulation, IL-6 blockade (e.g., tocilizumab), NLRP3 inhibition (e.g., MCC950), Sirtuin activation.	Immunometabolic reprogramming/ Inflammatory load.	Research tool/Preclinical/Hypothetical candidate strategy.	Lack of specific clinical evidence for perioperative AKI; inflammation is involved in both injury and repair; tocilizumab carries risks of immunosuppression and infection; MCC950 and Sirtuin activators are currently mainly in the preclinical stage.	Suitable for mechanistic validation and phenotype-enriched trials in populations enriched for inflammatory markers, but should not be recommended as routine treatment.	(203–206)
Time-windowed Multimodal Comprehensive Intervention	Preoperative risk optimization, correcting anemia/malnutrition/electrolyte disorders, maintaining intraoperative perfusion and oxygen delivery, postoperative KDIGO bundle, ERAS pathways, and early warning.	Comprehensive dimensions of the pathological triangle model.	Clinical routine/ Clinical adjunct, mechanistic integration pending verification.	Currently relies mostly on empirical management and comprehensive supportive therapy; prospective studies designed according to dominant imbalance dimensions are still lacking.	A currently more feasible practical pathway, emphasizing continuous management and dynamic adjustment from preoperative to intraoperative to postoperative periods.	(211, 212)
Bedside Scenario-based Stratified	Management Risk identification and monitoring focus adjustment based on scenarios such as sustained hypotension/blood loss, prolonged extracorporeal circulation/nephrotoxic exposure, diabetes mellitus/obesity/infection/chronic inflammation.	Comprehensive dimensions of the pathological triangle model.	Clinical adjunct/ Hypothetical stratification framework.	Not yet a validated diagnostic algorithm; the correspondence between different scenarios and specific phenotypes still requires prospective validation.	Used to assist clinical risk identification and research-based phenotype stratification, not to replace KDIGO diagnostic and treatment processes.	(214, 215)
Translation Pathway from Animal Models to Precision	Clinical Trials Establishing a phenotype classification system, conducting phenotype- enriched precision trials based on microcirculatory, mitochondrial, and inflammatory markers.	Comprehensive dimensions of the pathological triangle model.	Hypothesis generation/ Future research direction.	Insufficient ecological validity of animal models; mismatches in dosing timing, dose, endpoints, and enrolled populations often lead to translation failure; significant heterogeneity in AKI.	More suitable as a “mechanism–phenotyping–trial design” framework, rather than direct clinical treatment guidelines.	(216–219)

## 8 Future perspectives and research priorities

### 8.1 Level of evidence, controversies, and methodological limitations

Perioperative AKI often occurs in complex contexts involving low perfusion, ischemia-reperfusion, inflammatory activation, and metabolic stress. Although the “renal microcirculatory hypoxia–mitochondrial injury–immunometabolic reprogramming” pathological triangle model provides an integrative framework for understanding its pathogenesis, the dynamic sequence, relative weight, and dominant imbalance patterns of these three factors in real perioperative populations have not been fully validated (109). It should be emphasized that existing evidence does not uniformly support all inferences within the model. Although observational associations exist between perioperative hypotension, hemodilution, hyperoxia exposure, inflammatory response, and AKI risk, some hemodynamic optimization, individualized MAP management, oxygen supply regulation, anti-inflammatory/antioxidant strategies, and mitochondrial protective strategies have not consistently improved AKI outcomes. This suggests that interventions targeting a single pathway or a single macro-level indicator cannot adequately address the high heterogeneity of perioperative AKI. For example, achieving target MAP does not guarantee the restoration of renal medullary oxygenation; insufficient fluid resuscitation may lead to low perfusion, while excessive fluid administration may exacerbate venous congestion and renal interstitial edema; hyperoxia exposure may improve oxygen delivery but also increase oxidative stress risk. Therefore, the prevention and management of perioperative AKI cannot be simplistically equated with “raising blood pressure, increasing oxygen supply, or suppressing inflammation,” but should involve dynamic assessment of microcirculation, mitochondrial injury, and immunometabolic status. Furthermore, whether different surgical types and baseline populations correspond to distinct dominant imbalance patterns remains inadequately defined. In high-risk scenarios such as cardiopulmonary bypass cardiac surgery, major vascular surgery, and major abdominal surgery, the relative contributions of low perfusion, inflammatory response, and ischemia-reperfusion injury may differ (220). Conversely, elderly patients and those with comorbidities such as diabetes, hypertension, or metabolic disorders may have superimposed background factors including reduced renal reserve, microangiopathy, and chronic immune activation. Therefore, future research should clearly distinguish direct perioperative evidence, extrapolated evidence from other AKI scenarios, and preclinical mechanistic evidence, and further delineate the “pathological triangle model trajectories” for different surgical types and baseline populations, in order to identify more explanatory driving nodes and potentially intervenable pathways (**Table 4**).

**Table 4:** Level of evidence supporting key mechanisms in the pathological triangle model.

Mechanism module	Direct perioperative evidence	Main extrapolation evidence sources	Evidence assessment and recommendation
Renal Microcirculatory Hypoxia	Perioperative hypotension, hemodilution, extracorporeal circulation, transplantation, and high-risk surgery cohorts suggest an association with AKI risk; direct renal tissue oxygenation evidence is limited.	Shock, sepsis AKI, IRI, diabetic nephropathies, and CKD models.	Moderate evidence, partially dependent on extrapolation; recommended phrasing: “may be involved,” “indirectly supported,” “still requires perioperative validation.”
Mitochondrial Injury	Mitochondrial injury or metabolic abnormality signals observed in cardiac surgery, transplantation, nephrotoxic drug exposure, and perioperative AKI cohorts.	IRI, cisplatin AKI, sepsis AKI, PTEC models.	Strong mechanistic evidence, but insufficient perioperative chain-of-causation evidence; recommended phrasing: “may act as an intracellular amplifier.”
mtDAMPs and Immune activation	Perioperative trauma, extracorporeal circulation, blood transfusion, and reperfusion can be accompanied by the release of DAMPs and a systemic inflammatory response.	Sepsis, autoimmune diseases, IRI, systemic inflammation, and preclinical models.	Mostly indirect evidence; recommended phrasing: “candidate mechanism,” “may link mitochondrial injury to sterile inflammation.”
Immunometabolic Reprogramming	Diabetes mellitus, obesity, infections, chronic inflammation, and surgical stress are associated with AKI risk; direct immunometabolomic evidence is limited.	Sepsis AKI, IRI, ICU-AKI, inflammatory diseases, and immune cell metabolism studies.	Perioperative direct evidence is weak; recommended phrasing: “possible candidate amplification mechanism.”
Dominant Imbalance Phenotypes	Lack of validated perioperative classification standards and biomarker cutoffs.	AKI heterogeneity studies, biomarker subtypes, clinical risk models, and preclinical mechanism studies.	Hypothesis generation stage; recommended phrasing: “hypothetical candidate phenotypes.”
Targeted Intervention Strategies	Hemodynamic optimization, KDIGO care bundles, and ERAS pathways are supported by some clinical evidence.	Mitochondrial protection, NLRP3 inhibition, and AMPK/Sirtuin regulation, derived mostly from animal or cell studies.	Most are still mechanism-oriented candidate strategies and should not be directly equated with mature clinical recommendations.

### 8.2 Opportunities from advances in technology platforms and methodologies

New-generation technology platforms offer significant opportunities for elucidating the pathological triangle model. Single-cell transcriptomics, spatial omics, and metabolomics can delineate the lineage composition, metabolic status, and interactions of renal tubular epithelial cells, endothelial cells, and immune cells at higher resolution across different phases of AKI, thereby overcoming the limitations of traditional histology in complex mixed pathologies and dynamic evolution (194, 221). Molecular pathology platforms, such as the Molecular Microscope Diagnostic System (MMDx), also suggest that tissue molecular features may provide more refined cell–molecular annotations for perioperative AKI (222). At the population research level, perioperative databases and embedded study designs supported by Electronic Health Records (EHR) provide a practical foundation for introducing the pathological triangle model into risk prediction and phenotype clustering (223). In the future, mechanistic biomarkers such as NGAL, KIM-1, TIMP-2·IGFBP7, CCL14, U-mtDNA, and key metabolite profiles can be integrated with clinical variables to earlier identify AKI phenotypes driven by different pathologies and provide more rational enrollment strategies for phenotype-enriched trials. Meanwhile, biosensing and molecular imaging technologies, such as point-of-care molecular probe detection, photoacoustic imaging, and near-infrared imaging, also enable near-real-time monitoring of microcirculation, mitochondria, and immune-metabolic status (224, 225). However, these tools are still primarily in the research and early translation stages, and their standardization, reproducibility, and clinical accessibility require further validation.

### 8.3 Moving toward precision prevention and treatment: an individualized perioperative management framework based on the pathological triangle model

**Figure 6:**
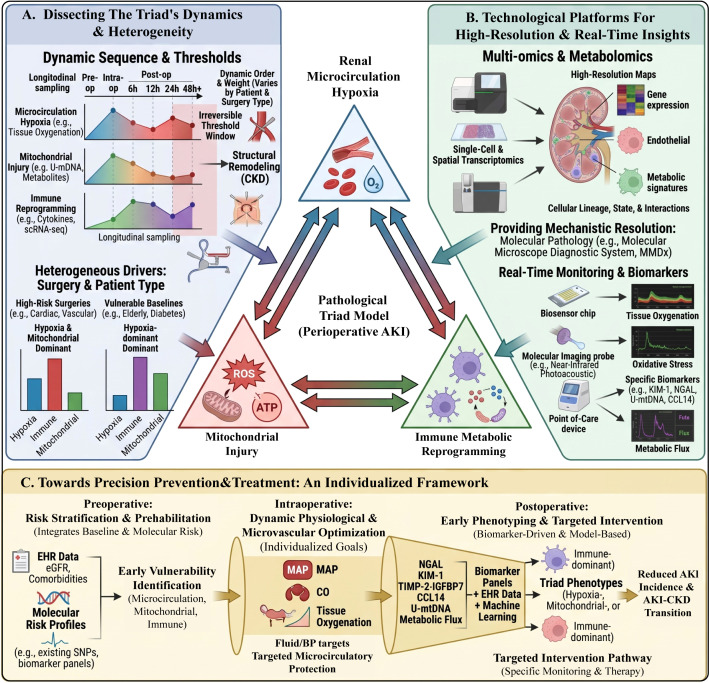
This figure presents the “pathological triad” framework for perioperative acute kidney injury (AKI) and its translation toward precision prevention and treatment. **(A)** highlights that renal microcirculatory hypoxia, mitochondrial injury, and immunometabolic reprogramming are not static parallel events, but dynamic, heterogeneous, and threshold-dependent processes shaped by surgical type and host vulnerability, jointly driving AKI progression toward structural remodeling and AKI-to-CKD transition. **(B)** illustrates the role of multi-omics, single-cell and spatial transcriptomics, real-time monitoring, and biomarker platforms in resolving triad imbalance, enabling identification of cellular states, molecular pathology, tissue oxygenation, oxidative stress, and metabolic flux changes. **(C)** further proposes an individualized intervention pathway: preoperative risk stratification and prehabilitation based on clinical and molecular profiles, intraoperative optimization centered on microcirculation and tissue oxygen delivery, and postoperative early phenotyping with biomarker panels, electronic health record data, and machine learning–assisted targeted intervention. Together, this framework aims to reduce AKI incidence and limit progression from AKI to chronic kidney disease.

In the future, a mechanism-enhanced risk assessment and decision support framework can be built around the pathological triangle model to drive the transition of perioperative AKI management from empirical, single-indicator identification to mechanism-driven and phenotype-oriented precision prevention and treatment. Unlike traditional risk identification that mainly relies on Scr and urine output, this framework emphasizes integrating patient baseline characteristics, intraoperative dynamic physiological data, and multidimensional biomarker information to assess the dominant imbalance link and stage in the three dimensions of “renal microcirculatory hypoxia–mitochondrial injury–immune metabolic reprogramming” (226, 227). In the preoperative phase, this framework can integrate eGFR, cardiac function, inflammatory and metabolic status, and, when necessary, molecular risk features to earlier identify high-risk patients with fragile microcirculation, insufficient mitochondrial reserve, or long-term immune activation, thereby prompting more intensive monitoring and prehabilitation measures. In the intraoperative phase, it can combine mean arterial pressure, cardiac output, volume responsiveness, oxygen delivery indicators, and available tissue oxygenation data to assist in identifying patients who, despite acceptable macro-parameters, already show trends of microcirculatory and oxygen supply abnormalities, supporting more individualized fluid and blood pressure management strategies (228, 229). In the postoperative phase, before a significant increase in Scr, it can identify high-risk trajectories by combining monitoring curves and mechanistic biomarkers, and correlate them with imbalance patterns such as microcirculation-dominant, mitochondria-dominant, or inflammation-dominant types, thereby supporting more targeted monitoring and intervention pathways (230). Therefore, future research should focus on validating whether this framework can reliably identify the dominant imbalance pattern of perioperative AKI and guide phenotype-enriched intervention trials (**Figure 6**).

## 9 Conclusion

Perioperative AKI is not merely a consequence of transient hypoperfusion but a cascading pathological process spanning multiple stages during and after surgery. The pathological triangle model of “renal microcirculatory hypoxia–mitochondrial injury–immune metabolic reprogramming” proposed in this paper does not posit microcirculatory dysfunction, mitochondrial injury, or immune inflammatory response as novel mechanisms, but rather integrates these widely studied mechanisms by synthesizing them within the specific framework of the perioperative AKI time window, multiple hits, and clinical heterogeneity. This model elucidates the heterogeneous onset, persistent progression, and risk of AKI-CKD transition in perioperative AKI, and postulates the existence of dominant imbalance patterns that can be prospectively validated. Compared with the traditional diagnostic system relying solely on Scr and urine output, the multidimensional stratification approach grounded in the pathological triangle model facilitates the integration of microcirculatory/tissue oxygenation assessment, biomarkers of mitochondrial injury, and immune metabolic readouts, providing a basis for mechanistic phenotyping, risk stratification, and phenotype-enriched trials. Notably, this model is currently primarily a framework for mechanism integration and hypothesis generation, rather than a mature clinical diagnostic tool; its true value lies in providing a more time-window- and stratification-oriented “pathological map” for future perioperative cohort studies, biomarker validation, and precision intervention trials.
